# A DC-Sensitive Video/Electrophysiology Monitoring Unit for Long-Term Continuous Study of Seizures and Seizure-Associated Spreading Depolarization in a Rat Model

**DOI:** 10.1523/ENEURO.0057-25.2025

**Published:** 2026-01-14

**Authors:** Jiayang Liu, Bruce J. Gluckman

**Affiliations:** ^1^Center for Neural Engineering, Pennsylvania State University, University Park, Pennsylvania 16802; ^2^Department of Engineering Science and Mechanics, Pennsylvania State University, University Park, Pennsylvania 16802; ^3^Department of Neurosurgery, Pennsylvania State College of Medicine, Hershey, Pennsylvania 16802; ^4^Department of Bioengineering, Pennsylvania State University, University Park, Pennsylvania 16802

**Keywords:** epilepsy monitoring unit, freely moving animal, long-term recording, spontaneous seizure, spreading depolarization, tissue oxygenation

## Abstract

There has been a long-term need for a low-cost, highly efficient, and high-fidelity epilepsy monitoring unit (EMU) suitable for synchronized multimodal home-cage monitoring of small-animal models of epilepsy and spreading depolarization. We present an accessible, scalable, highly space- and energy-efficient EMU capable of fulfilling chronic, continuous, synchronized, multiple-animal monitoring jobs. Each rig within the EMU can provide 16-channel high-fidelity, DC-sensitive biopotential recordings, head acceleration monitoring, voltammetry applications, and synchronized video recording on one freely moving rat. We present the overall EMU architecture design and subsystem details in each recording rig. We demonstrate long-term continuous in vivo recordings of spontaneous seizure and seizure-associated spreading depolarization from freely moving rats (male, 21; female, 6) prepared under the tetanus toxin model of temporal lobe epilepsy.

## Significance Statement

Long-term continuous DC-sensitive biopotential and video recordings are essential for capturing the dynamics of epileptic seizures and seizure-related spreading depolarizations, providing a deeper understanding of their underlying mechanisms. These recordings are invaluable for developing animal models of epilepsy, studying seizure prediction, drug testing, and investigating related neurological conditions such as mental health, aging, and dementia. They also reveal rare phenomena that short-duration recordings might miss. However, traditional methods are resource intensive. The new epilepsy monitoring unit introduced in this paper offers a cost-effective and space-saving solution for long-term high-fidelity, synchronized multimodal monitoring of freely moving animals, utilizing compact single-board computers and standard cages.

## Introduction

Epilepsy is the fourth most common neurological disorder in the United States after migraine, stroke, and Alzheimer's disease. It affects >2.2 million people in the United States and 65 million people worldwide (Hirtz et al., 2007; Institute of Medicine Committee on the Public Health Dimensions of the Epilepsies, 2012). Epilepsy is characterized by spontaneous recurrent seizures. Spreading depolarization/depression (SD) is a neurophysiological phenomenon characterized by abrupt changes in intracellular ion gradients and sustained neuronal depolarization, leading to loss of electrical activity, altered synaptic architecture, and vascular response. Electrophysiological features of SD recorded in humans closely resemble those observed in animal models (Cozzolino et al., 2018). Monitoring SD for research and neurointensive care in stroke and traumatic brain injury (TBI) patients (Lauritzen et al., 2011; Dreier and Reiffurth, 2015) poses methodological challenges in clinical neurophysiology (Hartings et al., 2017). SD can induce changes across slow and higher-frequency bands, and a hallmark of SD is a negative near-DC shift in the milli-Hertz range (<0.05 Hz), reflecting mass breakdown of electrochemical membrane gradients, with amplitudes reaching 35 mV or more (Leao, 1947; Collewijn and Harreveld, 1966; Hansen and Zeuthen, 1981; Canals et al., 2005).

Long-term continuous recordings that include multiple modalities, such as biopotential and video recordings, are essential for studying epileptic seizures in small, freely moving animals. These recordings are crucial for addressing various epilepsy-related questions, including the development of animal models of epilepsy (Bertram and Cornett, 1993; Jefferys and Walker, 2006; Raedt et al., 2009), understanding epilepsy physiological mechanism (Walter et al., 1951; Worrell et al., 2008; Jiruska et al., 2010), and the study of seizure prediction and forecasting (Cook et al., 2013; Kingwell, 2013; Wang et al., 2021; Xiong et al., 2021; Ibrahim et al., 2022; Liang et al., 2022). These recordings can also aid in drug testing and in elucidating the mechanisms of epileptogenesis and the maturation of epilepsy (Watanabe et al., 2017).

Additionally, DC-sensitive, high-resolution, and wide dynamic range chronic multi-odal recordings—such as electroencephalogram (EEG) video and synchronized electrocardiogram (ECG)—can be utilized to investigate various aspects related to neurological health, including SD, pharmacology, epidemiology, TBI, and sudden unexpected death in epilepsy (SUDEP). Furthermore, these recordings can help explore the comorbidity and interactions with other neurological disorders, such as mental health issues, aging, dementia (including Alzheimer's disease and Parkinson's disease), and sleep disorders. Importantly, long-term continuous recordings help capture the dynamics of epilepsy that are associated with rare events, revealing phenomena that cannot be appreciated with short-duration recordings. The need for exhaustive continuous recording can also be understood by considering that the likelihood of a false-negative diagnosis of epilepsy increases inversely with the base seizure rate, yet the ability to do such classification and to identify the mechanisms surrounding changes in seizure susceptibility is critical for the development of clinically relevant treatments. Long-term, continuous multimodal recordings on multiple animals are typically resource intensive, taking a sizeable space and multiple computers.

In this paper, we introduce a new epilepsy monitoring unit (EMU) designed for long-term, high-fidelity, multimodal home-cage recordings in freely moving animals. The system was developed from the need for continuous monitoring of both electrophysiological and behavioral signals during the normal state of vigilance (SOV), spontaneous seizures, and seizure-associated SDs.

From the electrophysiology [local field potentials (LFPs) or electrocorticography (ECoG)] standpoint, the EMU design needs to balance the requirements for detecting seizures and reliably characterizing SDs. Seizure discharges often span a broad frequency range, from slow rhythms (<1 Hz) to fast oscillations (>200 Hz), demanding high sampling rates (e.g., 1 kHz) and a wide bandwidth to capture both initiation and evolution of ictal discharges (Schevon et al., 2008; Buzsaki et al., 2012). In contrast, SDs are characterized by large, slow, negative DC shifts that last tens of seconds, which require DC-sensitive recording to avoid signal distortion (Dreier, 2011; Hartings et al., 2011). These DC shifts can reach tens of millivolts in amplitude (Leao, 1947; Collewijn and Harreveld, 1966; Hansen and Zeuthen, 1981; Canals et al., 2005), far greater than typical LFP/ECoG signals (50–150 µV), making a wide dynamic range essential to prevent amplifier saturation while maintaining sensitivity to small fluctuations. To ensure high-fidelity measurement of both ultralow-frequency and small-amplitude signals, high input impedance (much higher than electrode impedance) is also required to minimize electrode polarization artifacts and signal loss. Finally, high-resolution analog-to-digital conversion (≥18 bits) is necessary to capture both microvolt-level oscillations and tens of millivolt-scale depolarizations within the same recording channel. Multichannel coverage is additionally required to track the spatiotemporal propagation of SDs across brain regions (Dreier and Reiffurth, 2015).

Behavioral monitoring was incorporated to complement electrophysiology. Video synchronized with LFP/ECoG recordings allows correlation of electrographic events with seizure semiology, and head acceleration measurements provide an additional measure of motor manifestations and postictal states, as well as state score (Sunderam et al., 2007).

The EMU also incorporates several additional features critical for long-term monitoring of freely moving animals. Electrochemical sensing capabilities extend monitoring beyond electrical activity to include local tissue chemistry, providing complementary insight into metabolic and ionic dynamics during seizures and SDs. A robust but flexible commutator cabling system enables high-speed data transmission while allowing animals to move freely. The commutator cabling system also ensures cables can be quickly replaced if damaged, minimizing recording downtime. The system is scalable, supporting both single-animal and cohort studies with multiple animals. Long-term, uninterrupted monitoring is supported by continuous power supply, streamlined data handling, and seamless integration with ventilated cage systems. Importantly, the design allows routine animal handling without interrupting ongoing recordings, ensuring data continuity across weeks of observation. Together, these features establish a comprehensive, low-cost, and space-efficient platform for detecting and characterizing the complex interplay between seizures and SDs in freely moving animals.

## Materials and Methods

**Figure 1.**
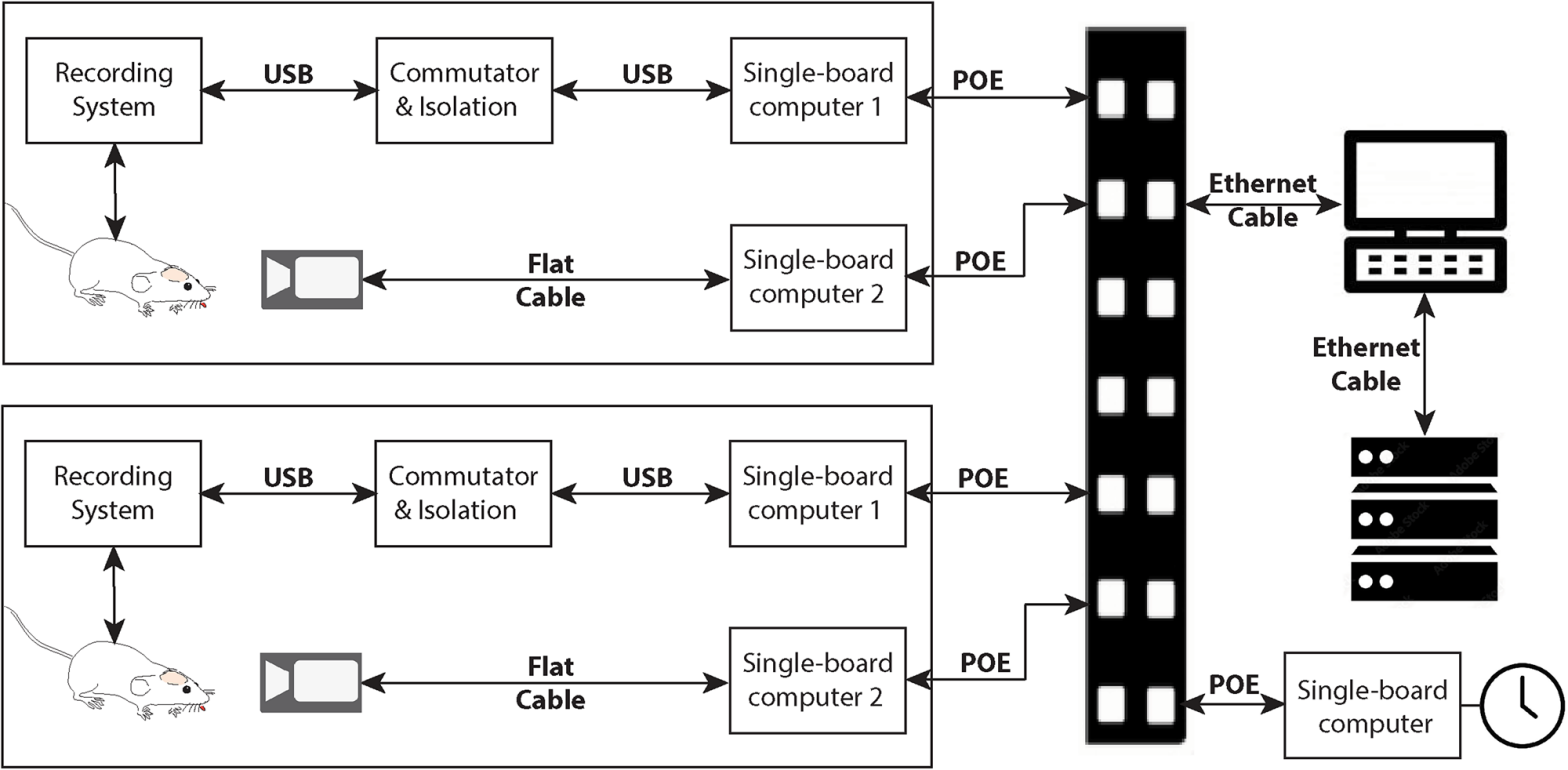
EMU schematic. Two rigs are shown here as a demonstration. In each rig, a recording system mounted on the rat's head connects to a single-board computer via a USB cable and a commutator and isolation system. A low-light-level compatible camera connects to another single-board computer via a flat cable. Both single-board computers are fixed to the cage dome and continuously acquire data to a NAS through PoE cables and a switch. A time server is used for synchronization across all rigs.

The EMU schematic shown in Figure 1 features two rigs as a demonstration with the option to add more as needed. Within each rig, via a USB cable and a commutator and isolation system, a DC-sensitive recording system is connected to a single-board computer, which is fixed to the cage dome. The single-board computer continuously collects data such as LFP, ECoG, ECG, and head acceleration to a network-attached storage (NAS) via an Ethernet cable and a power over Ethernet (POE)-supported switch. A separate low-light-level compatible camera system, also fixed to the cage dome, spools video recordings to the same NAS through another Ethernet cable and the same switch. All single-board computers are time-synchronized and communicate with the control station sitting outside the animal facility. Each rig fits into a standard commercial ventilated cage rack with a standard autoclave-ready cage base, meaning the EMU can be directly adapted without extra components. To assist with husbandry and cage sanitation, while minimizing direct animal contact, we have further implemented a touch-free cage-cleaning method that does not interrupt recordings, which is especially important for epileptic animals that tend to be skittish.

### Recording system hardware design

**Figure 2.**
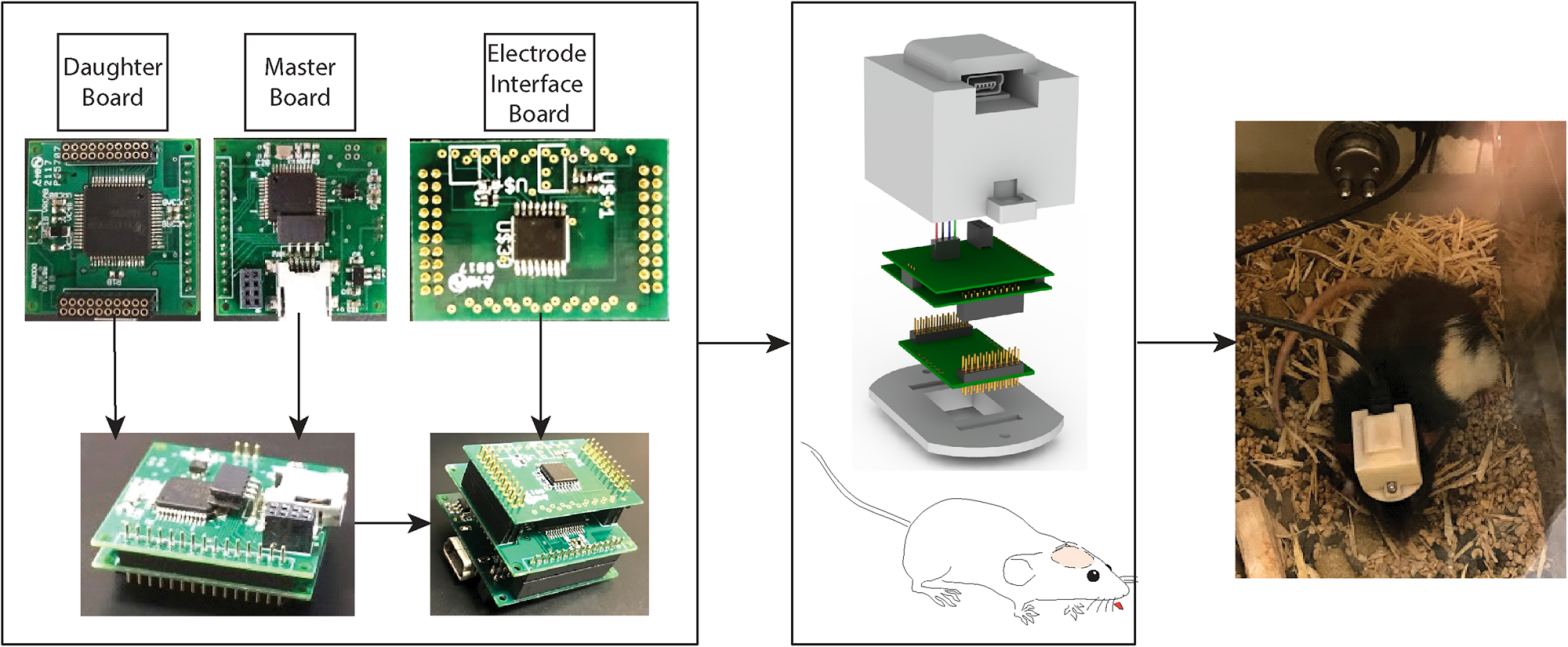
Recording system arrangement. The recording system in each rig consists of a master board, a daughter board, and an EIB, arranged from top to bottom. A 3D-printed box with a cover and a plate holds the three boards. The cover includes a mini-USB connector for connecting the USB cable. The plate secures the EIB and will be cemented to the animal's head during the surgical process. The master board and daughter board are soldered together and can be plugged in or removed from the EIB via interconnectors.

The current design is adapted from earlier high-performance, low-cost, DC-sensitive recording systems suitable for recording from human beings (Jain et al., 2011) or chronically from mice (Ssentongo et al., 2017) that utilize commercial off-the-shelf integrated components. We demonstrate here a design capable of fulfilling long-term synchronized multimodal monitoring for freely moving rats. The system has 16 DC-sensitive recording channels with a sampling rate up to 16 kHz, an accelerometer for head acceleration measurement, and an electrochemical instrument (EI). Subsystems arrangement within the recording system has also been improved into a three-layer stack structure and can fit into a 3D-printed box (1 × 1 × 1 in). This box is small and light enough to be fixed to a rat's head as shown in Figure 2. From top to bottom, the three layers are the master board, the daughter board, and the electrode interface board (EIB). The three boards connect via interconnects: the master board and daughter board are soldered together, and this pair can be plugged in or unplugged from the EIB. The EIB, described later, becomes part of the head-attached construct during surgery. This design greatly facilitates experimental implantation and gives flexibility for different recording scenarios.

The master board contains a microcontroller, power regulation subsystem, two system-embedded peripherals [a tri-axis accelerometer and a digital-to-analog converter (DAC)], and a bus to connect to additional peripherals [analog front-end (AFE) amplifiers] on the daughter board.

The microcontroller (Texas Instruments, MSP430 series) is responsible for writing to or reading from these peripherals and communicating with the controlling computer via a USB connection. Biopotential recordings are accomplished through two AFE amplifiers (Texas Instruments, *ADS1299*). The ADS1299 is a high-performance eight-channel, simultaneous-sampling, 24 bit continuous-time delta–sigma analog-to-digital converter (ADC) with a built-in programmable gain amplifier featuring low noise and high impedance input. The recording system thus can provide 16 channels of biopotential recordings with submicrovolt digitization resolution and ±4.5 V dynamic range. Head acceleration is measured using the accelerometer (STMicroelectronics *LIS2HH12*) as a complementary signal for behavioral SOV scoring (Sunderam et al., 2007). A DAC (Analog Devices *LTC2642*) is included on the master board and serves with the microcontroller as a function generator for peripheral control. The power regulation subsystem is built on the master board. It receives +5 V input through the USB connection and supplies regulated +3.3 V for digital and +2.5 V/−2.5 V for analog power.

The EIB behaves as an interface between implanted electrodes and the recording system. Electrode leads were connected to the EIB in a riveting way with electrode attachment pins, providing an easy and reliable long-term connection (Neuralynx). The EIB can be plugged in or unplugged from the system via interconnectors. In practice, the EIB is permanently cemented to the animal head during surgery, allowing the master and daughter boards to be plugged in and connected to all electrodes. After recording, the EIB can be disposed of, and the master and daughter boards can be reused.

**Figure 3.**
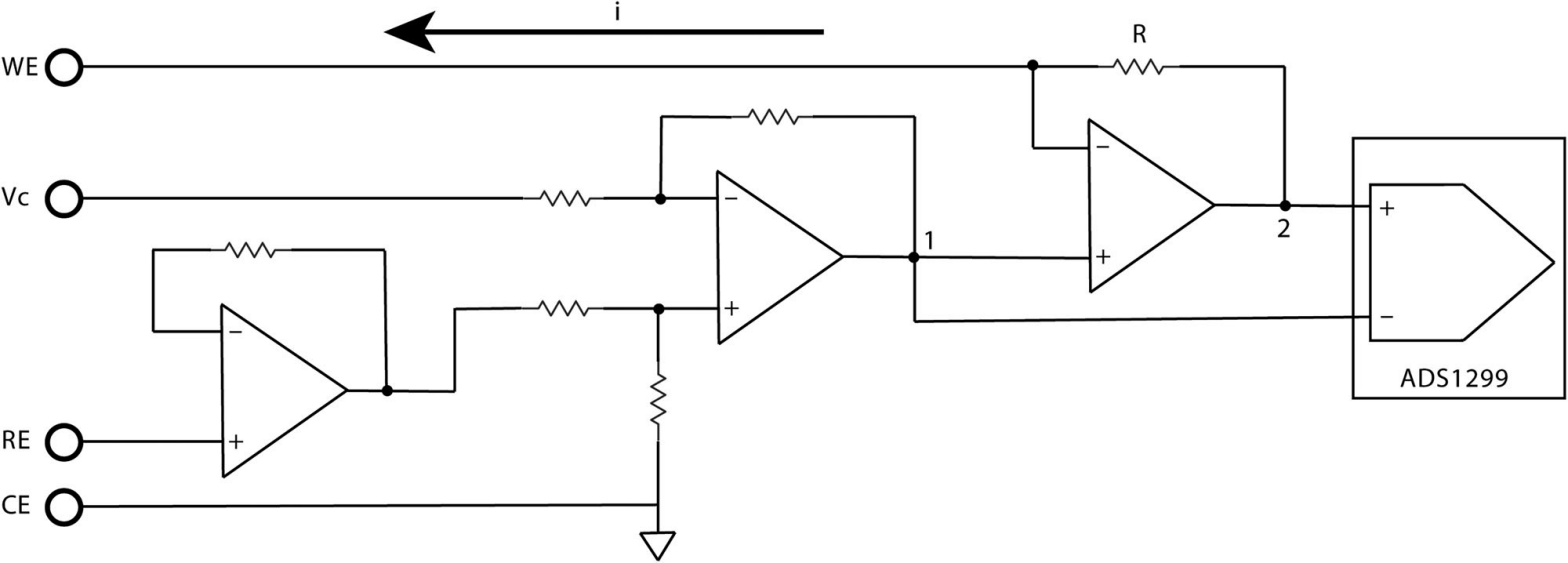
Floating three-electrode potentiostat circuit. Compared with a classic potentiostat setup, in the floating three-electrode potentiostat circuit, the CE is grounded. The RE potential is allowed to float, and the current is driven through the WE to enforce its potential relative to the RE. The current (i) is calculated using the potential difference between Points 1 and 2, divided by the resistance (R). The potential difference is recorded by one channel of ADS1299, and the applied control overpotential (Vc) is provided by the DAC (LTC2642).

A floating three-electrode potentiostat circuit was built on the EIB, as shown in Figure 3. Classic potentiostat circuit designs lock the working electrode (WE) at zero potential (ground) of the power supply and drive current to the counter electrode (CE) to force the reference electrode (RE) to some programmed value. In the floating potentiostat design, the CE was grounded. The RE potential was allowed to float with respect to the amplifier power supply, and the current was driven through the WE to enforce its potential with respect to the RE. The current (1) was calculated using the potential difference between circuit Points 1 and 2 of Figure 3, divided by the resistance (R). The potential difference was recorded by one channel of ADS1299, and the applied control overpotential (Vc) was provided by the DAC (LTC2642) in the function generator subsystem of the master board. The three-electrode potentiostat circuit, combined with the function generator, when appropriately connected to electrodes, produces an EI suitable for a range of electrochemical applications.

### Recording system firmware and software design

The embedded firmware in the recording system was programmed based on the MSP Driver Library and USB API, using programming language C (ISO/IEC 9899:2018 standard), compiled under Microsoft Windows 11 (Microsoft), and downloaded into the MSP430 through a custom-made JTAG port on the master board via MSP430 USB-Debug-Interface MSP-FET430UIF Programmer Debugger Emulator.

**Figure 4.**
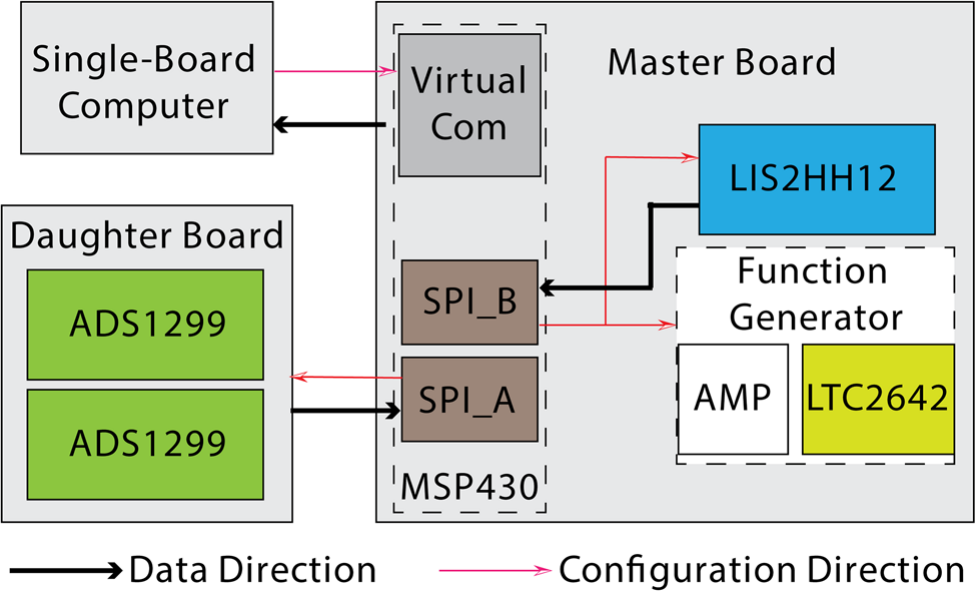
Recording system firmware design. In the firmware design, the microcontroller (MSP430) on the master board interfaces with peripheral elements through the universal serial communication interface set to the SPI mode. Specifically, it uses SPI_A with the analog front ends (AFEs) of ADS1299s on the daughter board and SPI_B with the accelerometer (LIS2HH12) and the DAC (LTC2642) in the function generator. The microcontroller communicates with the single-board computer via the Texas Instruments USB to virtual COM port. The microcontroller receives commands from and sends recorded data packets to the single-board computer through a USB connection. Each data packet consists of 63 bytes, including a 3 byte counter, 3 bytes for the status of ADS1299 #1, 24 bytes for ADS1299 #1’s eight-channel data, 3 bytes for the status of ADS1299 #2, 24 bytes for ADS1299 #2’s eight-channel data, and 6 bytes for the LIS2HH12’s acceleration data.

The firmware incorporated several functional blocks as shown in Figure 4. The microcontroller (*MSP430*) interfaced with the AFEs (*ADS1299*), the accelerometer (*LIS2HH12*), and the DAC (*LTC2642*) via the universal serial communication interface in synchronous peripheral interface (SPI) mode. Specifically, it interfaced with the analog front ends (AFEs) of ADS1299s on the daughter board via SPI_A and with the accelerometer (LIS2HH12) and the DAC (LTC2642) in the function generator via SPI_B. The microcontroller communicated with the single-board computer via the Texas Instruments USB to virtual COM port. Data from two *ADS1299s* and an *LIS2HH12* were simultaneously transmitted to *MSP430*, while configuration inputs from *MSP430* were received by *ADS1299*, *LIS2HH12*, and *LTC2642*, allowing for uninterrupted recording updates. The microcontroller communicated with the single-board computer via USB using the Texas Instruments USB to virtual COM port driver firmware. The microcontroller received commands from and sent recorded data packets to the single-board computer via the USB connection. Each data package consists of 63 bytes including 3 bytes-counter, 3 bytes-status of *ADS1299#1*, 24 bytes *ADS1299#1’s* eight-channel data, 3 bytes-status of *ADS1299#2*, 24 bytes *ADS1299#2’s* eight-channel data, and 6 bytes *LIS2HH12’s* acceleration data. Data stream sending operation occurred in the “background” whenever the USB bus was available until it was completed to take full advantage of background operation. The function generator in EI was implemented based on a DAC, *LTC2642*. We implemented five waveforms for multiple voltammetry applications: constant potential amperometry (CPA), fast-scan cyclic voltammetry, long-term pulse voltammetry, differential pulse voltammetry, and sinusoidal coupled with DC potential.

**Figure 5.**
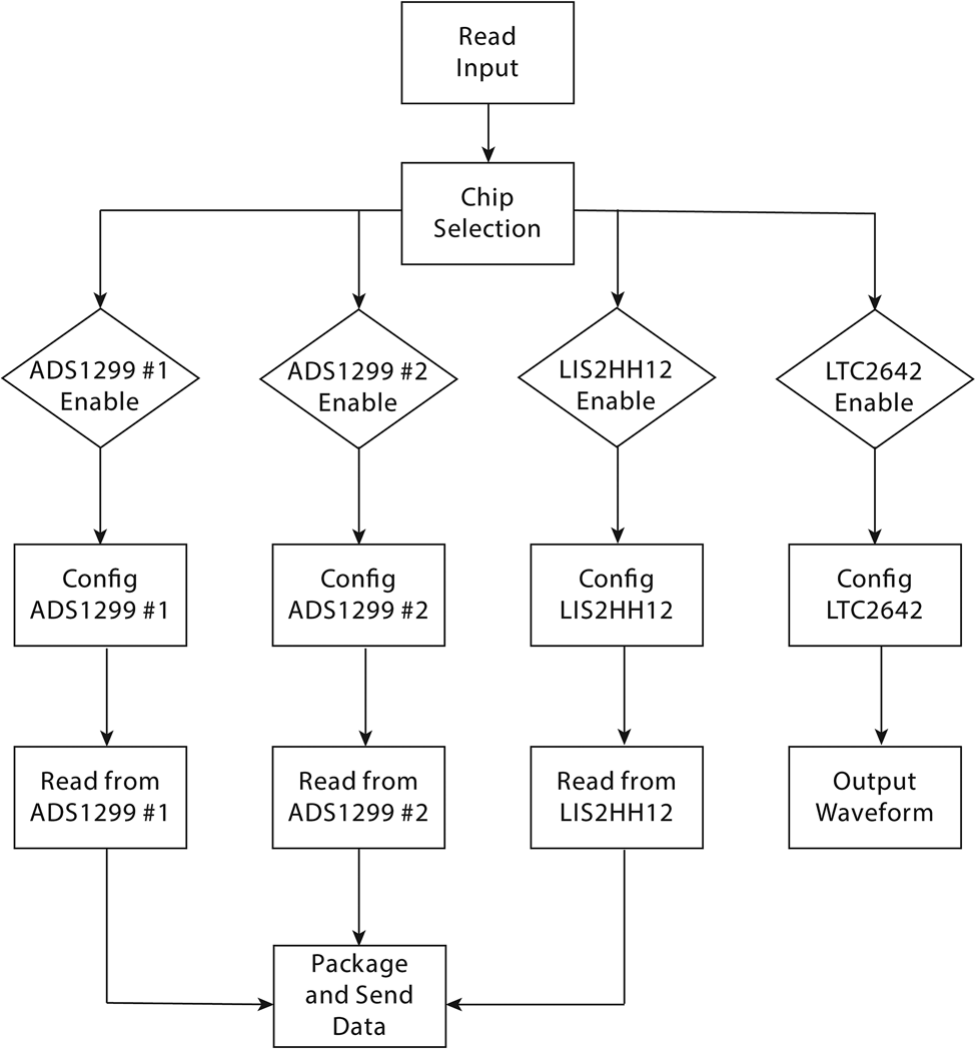
Software for data acquisition and process. The software for data acquisition and processing on the single-board computer is coded in Python (Python Software Foundation) under Linux (Linus Torvalds). It includes recording system configuration input file (Read Input), data reading and processing, and data transmission to the NAS. The input file contains configuration details (Config.) for all peripheral elements, including two ADS1299 AFEs, the accelerometer (LIS2HH12), and the DAC (LTC2642). Configuration details can be edited at the control station. Data packets are continuously recorded and transmitted to the NAS over an Ethernet cable, where they are stored in binary files. Each file begins every hour, with a 4,096 byte header that includes the configuration details.

The data acquisition and process software in the single-board computer was coded using Python (Python Software Foundation) under Linux (Linus Torvalds), including recording system configuration (Read Input), data reading and processing, and data sending to the NAS as shown in Figure 5. Input configuration details, such as parameters of two *ADS1299* AFEs, the accelerometer (*LIS2HH12*), and the DAC (*LTC2642*), can be edited at the control station. Data packets are continuously recorded and transmitted to the NAS over an Ethernet cable, where they are stored in binary files. Each file begins every hour, with the first 4,096 bytes containing a header that includes the configuration details. Beginning at the 4,097 byte, data are stored in 63 byte packets as described above until the 1 h limit is reached.

### Commutator and isolation system

**Figure 6.**
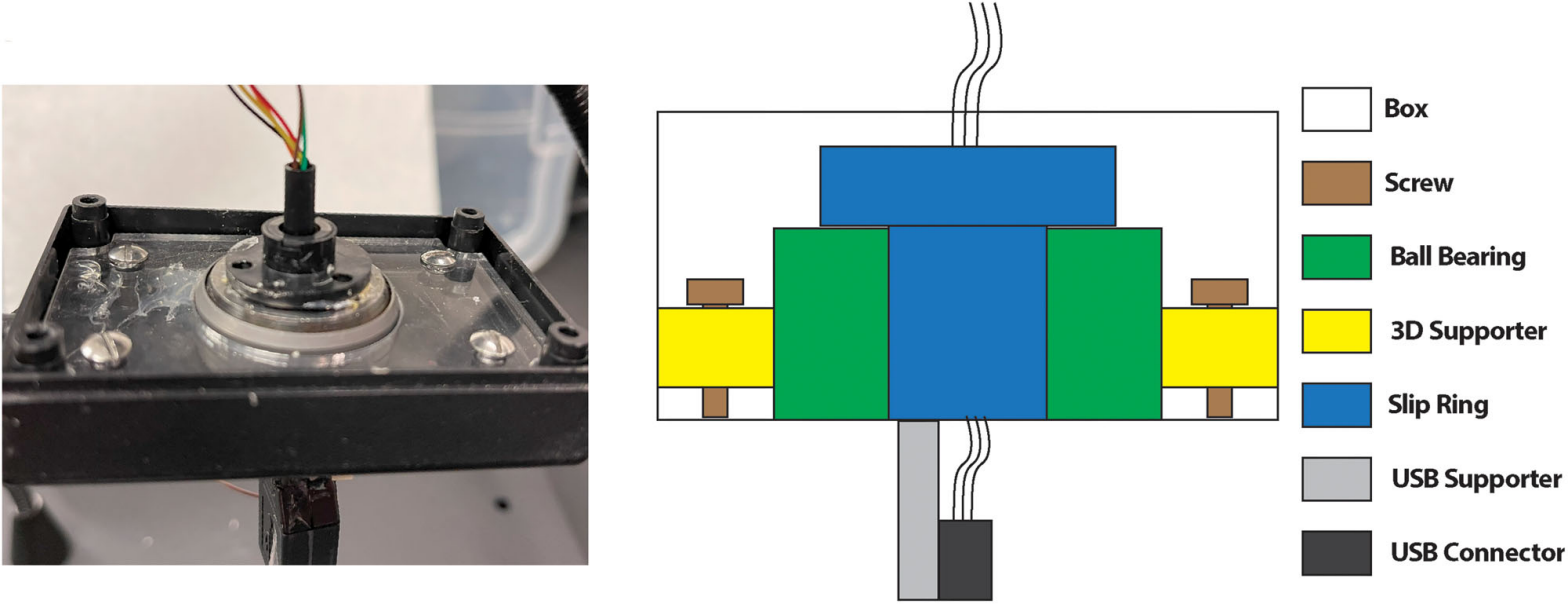
Commutator design. The commutator provides a USB 2.0 connection (USB connector) to the recording system and primarily consists of a slip ring (Moog's Components Group) and a thrust ball bearing (McMaster-CARR 60715K11). The thrust ball bearing is glued to the slip ring. A 3D-printed mechanical supporter (3D supporter) holds both the slip ring and the thrust ball bearing and is fixed in a box.

Each rig within the EMU can fulfill chronic continuous recording from a freely moving rat using a commutator and isolation system connected to the recording system via a USB cable. As shown in Figure 6, the commutator provides a USB 2.0 connection and was built with a slip ring (Moog's Components Group) and a thrust ball bearing (McMaster-CARR 60715K11). The thrust ball bearing, glued with the slip ring box, supports the axial forces from the USB cable weight or various movements from the animal. The design protects the slip ring commutator, whose function is sensitive to misalignment and whose bearings are designed to support transverse forces only. The slip ring has six 2A/120VAC circuits, and we used four of them for the USB connection. A 3D-printed mechanical supporter was used to hold the slip ring and the thrust ball bearing. The whole structure fits in a box. The USB supporter eliminates the vertical tension on wires.

The recording system communicates with the single-board computer and is also powered by the single-board computer. The recording system sits on the animal's head and thus also electrically contacts the animal, which potentially exposes the animal to the risk of electrical shock, burns, and damage directly due to leakage current resulting from improper grounding and electrical isolation (Been, 2007). To alleviate these risks, we utilized an electrical isolation system that minimizes transmission of 120 V/60 Hz to the animal. The isolation system helps to minimize pickup of line noise by minimizing the charge needed to tightly couple the amplifier power supply and the subject, which is mediated by current passing through the ground electrode and therefore minimizes common-mode signals. The isolation system incorporates a full/low-speed 5 kV USB2.0 compatible digital isolation chip and a 5 kV isolated DC-to-DC power converter (Analog Devices *ADuM4160* and *ADuM6000*). The *ADuM4160* provides mechanisms for detecting the direction of the USB data flow and controlling the state of the output buffers. The design of this system meets both creepage and clearance distance criteria in the reinforced isolation type defined in Standard IEC (International Electrotechnical Commission) 60601-1 (Been, 2007), which defines medical-equipment electrical–safety conditions necessary to protect patients, operators, and the surroundings.

### Video recording system

**Figure 7.**
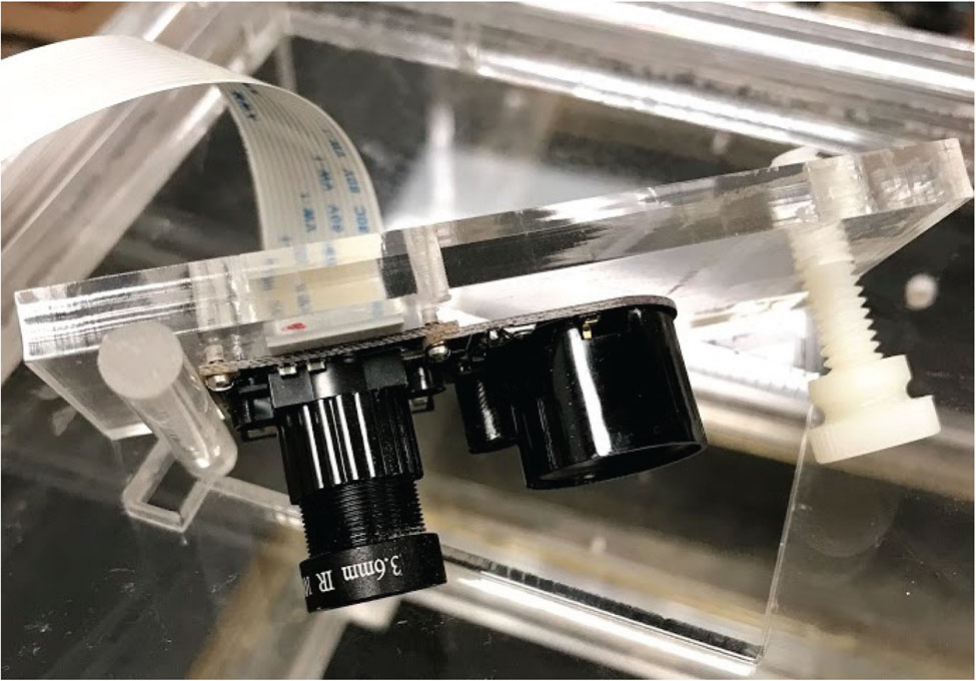
Low-light-level compatible video recording system. The video recording system includes a 5 megapixel, fixed-focus lens infrared night vision surveillance camera capable of providing 1,080p video recordings at 30 fps. It also features a 3 Watt infrared light lamp that enables 24 h continuous recording, a mechanical support structure that can be adjusted to change the video recording coverage, and a single-board computer (data not shown) for configuring video parameters and recording video files. The camera is connected to the single-board computer via a flexible flat cable.

In addition to biopotential and acceleration recordings, a synchronized 24 h continuous video recording of epileptic animals offers behavioral assessment during seizure events. We have built a low-light-level compatible video recording system, as shown in Figure 7. The video recording system includes a 5 megapixel, fixed-focus lens infrared night vision surveillance camera, which can provide 1,080 p at 30 fps video recordings, a 3 W infrared light lamp that enables a 24 h continuous recording, a mechanical supporting structure that can be adjusted to change the video recording coverage, and a single-board computer for video parameters configuration and video file recordings. The camera is connected to the single-board computer via a flexible flat cable. The single-board computer, fixed to the cage dome, continuously sends recorded h264 video files to the NAS, initiating new files hourly.

### Single-board computers and local area network

Commercial products for animal biopotential and video recordings usually involve the use of a desktop or laptop with specifically designed software for data retrieval and processing. In our EMU, we implement one of the most popular single-board computers, the Raspberry Pi 3 (RPi3; Raspberry Pi Foundation). Compared with desktops or laptops, the RPi is low-cost and generates much less heat and noise. It also features an extremely small size (3.7″ × 2.5″ × 1.0″) and can be easily fixed or removed from the dome of the cage without occupying extra space in the animal room. The RPi3 has four USB ports, an Ethernet port, a quad-core 1.2 GHz Broadcom BCM2837 64 bit CPU, and 1GB RAM. It also has a CSI camera port for connecting a Raspberry Pi-compatible camera. All the software and hardware connections are easily adapted to future versions, e.g., RPi5.

Each rig can be duplicated for multiple-animal monitoring applications. We set up a private local area network (LAN) with a control station, a NAS (Drobo B810n, Drobo storage company), a POE-supported switch (NETGEAR-FS726TP, NETGEAR), a time server, and all the other RPi3 used in the EMU, as shown in Figure 1. On the control station, we can access the data on NAS through the application graphic user interface and remotely communicate with each PRi3 through remote applications, e.g., Putty. For data protection purposes, the LAN is in an “offline” state, and no RPi3 within the LAN can access or be accessed from the Internet. An additional RPi3 is equipped with a real-time clock module (PCF8523, Adafruit Industries) and set as the time server of the LAN. All other RPi3s are synchronized with this server as time clients through Network Time Protocol. All RPi3s were set using a shell script to follow the same startup process after being connected to the switch through POE cables. The setup process is to automount on the NAS, get time-synchronized with the time server, read in the input file from the control station, auto-start recording data or video, and send data to the NAS.

### Built-in EI and in vitro oxygen sensor calibration

The built-in EI in each rig extends our EMU's application scenario, such as constant voltage amperometry (CPA) applications for tissue-level oxygen concentration monitoring. We conducted in vitro CPA oxygen sensing for calibration purposes to demonstrate the capabilities of the built-in EI. We implemented a three-electrode electrochemical cell connected to the EI. The setup includes a WE made of platinum (Pt) wire, an Ag/AgCl pellet RE, and a CE, which is a Pt plate. During the experiment, we determined the oxygen content by admixing two separate phosphate-buffered saline (PBS) solutions: one solution was saturated with air, achieved by bubbling air through it; the other was nearly devoid of oxygen, created by bubbling nitrogen (N2) through it. The three electrodes were submerged in the air-saturated solution, and N2-saturated solution was added in discrete known amounts to dilute the overall oxygen concentration. The experiment was conducted at room temperature, and the pH of the PBS solution was measured at 7.48 using a pH meter (Model P771, Anaheim Scientific).

**Figure 8.**
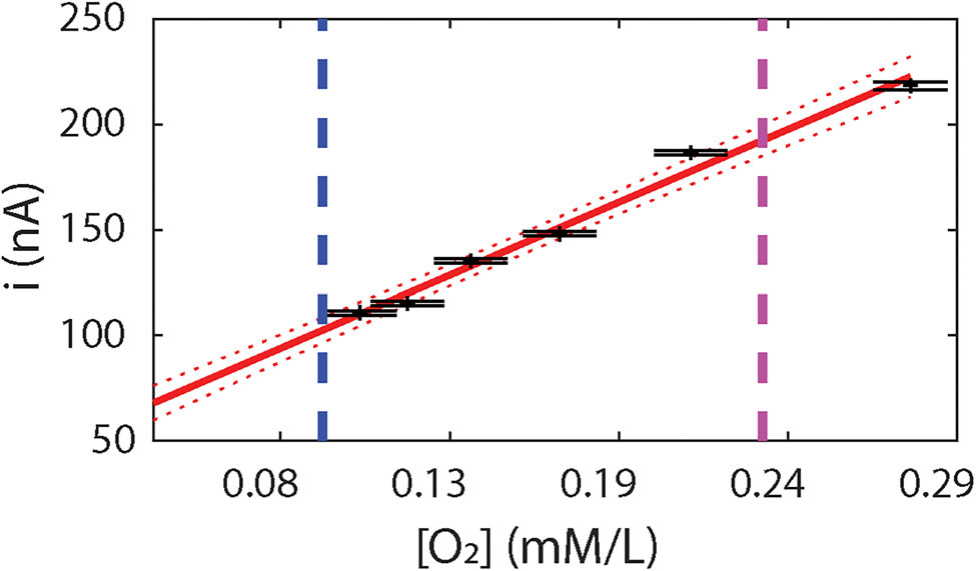
CPA recording for oxygen sensing electrode calibration. A linear fitting result of the CPA recording for oxygen sensing electrode calibration is presented, where the *X*-axis represents the oxygen concentration (mM/L) and the *Y*-axis shows the response current (nanoamperes). Each data point is derived from the addition of N2-saturated PBS solution. For each addition, we recorded data for ∼5 min, and each value was calculated from the middle 80 s of this recording to eliminate any transients associated with the addition process.

The resulting oxygen concentration was derived from standard stoichiometric calculations. We performed CPA measurements by applying a bias potential of −0.65 V on the WE with respect to the RE. For each data point, we added a fixed volume of N2-saturated PBS solution. After each addition, we recorded data for ∼5 min, and the reported value was calculated using the middle 80 s of this 5 min recording, excluding any transient responses associated with the addition of the N2-saturated PBS solution. The CPA calibration result is shown in Figure 8, using units millimole per liter (mM/L). In the brain tissue, the oxygen concentration, the partial pressure of oxygen (*PO*_2_), and the oxygen tension are mutually related values that, in principle, can be derived from one another. The oxygen concentration expressed in moles per liter (mol/L) is used to measure dissolved oxygen. The *PO*_2_ reflects the amount of free oxygen molecules and equals the pressure that oxygen would exert if it occupied the space by itself (Subczynski and Swartz, 2005). The calibration is well approximated by a linear fit over the physiological range from ∼0.09 mM/L (the tissue oxygen concentrations) to ∼0.23 mM/L (the arterial oxygen concentration; Ortiz-Prado et al., 2019), marked by the purple and blue lines.

### Animal surgery and care

All experimental protocols were approved by the Institutional Animal Care and Use Committee. We used Long–Evans rats (21 males and 6 females) obtained from Charles River Laboratories or ENVIGO, weighing between 250 and 500 g.

For LFP recordings in the hippocampus (four rats had LFP recordings in the thalamus or medial septum), we employed 50 µm microreaction chamber (µRC) electrodes. The µRC electrode incorporates an internal electrolyte reservoir and a greatly expanded effective electrochemical surface area, providing a low-impedance interface that suppresses polarization by allowing charge exchanged through reversible faradaic reactions within its microreaction chamber rather than accumulating at the tissue–metal boundary (Shanmugasundaram and Gluckman, 2011). Two µRC electrodes with tips spaced 125–250 µm apart dorsally were used as bipolar pairs, both referenced to the same screw electrode. This configuration greatly cancels common-mode drift from the screw reference, improving DC stability and enabling reliable measurement of local DC shifts. When paired with the ADS1299, this setup allows stable recording of slow and ultralow-frequency signals in the hippocampus, supporting characterization of both seizure activity and seizure-related SD events.

For ECoG recordings in the cortex, screw electrodes were utilized. For ECG recordings, lead electrodes were employed, and for tissue oxygen measurements using CPA, we used a 250-μm-diameter platinum (Pt) wire as the WE and a custom-made Ag/AgCl electrode as the RE. The Ag/AgCl electrode was created by inserting a 100-µm-diameter silver wire into a 155-µm-diameter polyamide tube filled with Ag/AgCl ink (CI-4001 Silver/Silver Chloride/Vinyl, Nagase America). It is important to note that Ag/AgCl electrodes are typically not used for direct tissue penetration due to their potential cytotoxicity (Robinson, 1968; Geddes, 1972). In our demonstration, the Ag/AgCl electrode is utilized solely as a RE in the cortex, ensuring minimal contact with the brain and reducing the risk of toxicity. Similar applications have been implemented in commercial electrochemical sensors targeted at the neuroscience community (e.g., Pinnacle Technology).

**Figure 9.**
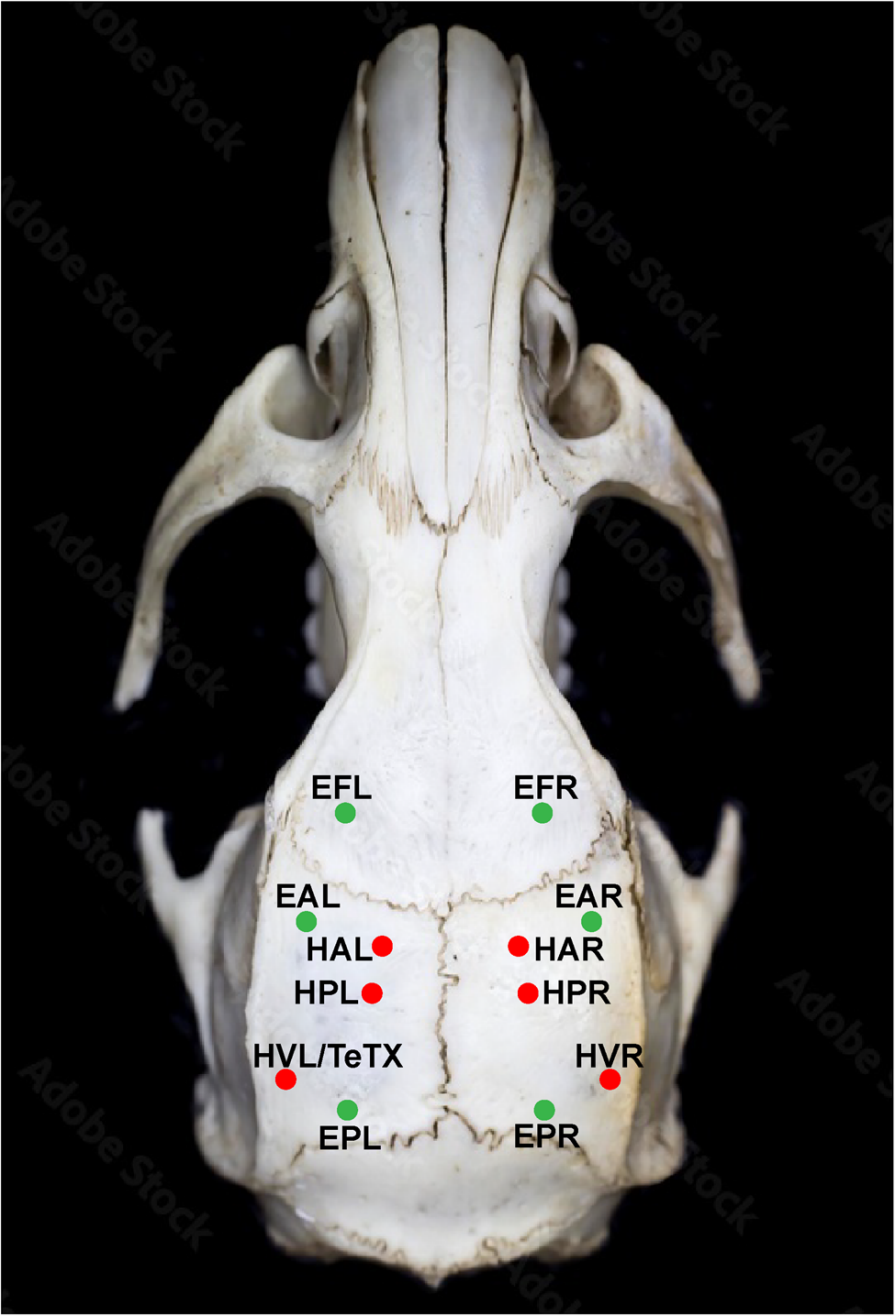
Stereotaxic coordinates of electrodes. All coordinates were bregma-referenced (AP, ML, DV). Red dots show coordinates of depth electrodes for LFP measurements in the hippocampal. HAL/R (hippocampal anterior left/right): −2.5 mm, ±2.0 mm, −3.2 mm. HPL/R (hippocampal posterior left/right): −3.9 mm, ±2.2 mm, −2.88 mm. HVL/R (hippocampal ventral left/right): −5.51 mm, ±5.35 mm, −6.1 mm. Green dots show coordinates of stainless steel screw electrodes measuring ECoG. EFL/R (EcoG for frontal left/right cortex): 2 mm, ±3 mm. EAL/R (EcoG for anterior left/right cortex): 1.5 mm, ±4 mm. EPL/R (EcoG for posterior left/right cortex): −6.5 mm, ±4 mm. The toxin injection site is at HVL/TeTX before electrode implantation to minimize additional cortical damage. For oxygen sensing, the WE is implanted at HAL and the RE at HVL after toxin injection. Skull figure adopted from Зыков (n.d.).

All surgeries were conducted under deep anesthesia using ketamine (90 mg/kg) and xylazine (15 mg/kg). A heating pad was utilized under the animal to maintain a constant body temperature of 37°C. Rats were secured in a stereotaxic frame with ear bars and given preoperative Buprenorphine Ethiqa XR (3.25 mg/kg) for pain relief. Lidocaine (<5 mg/kg) was injected subcutaneously at the incision site. Burr holes were drilled with an electric drill based on stereotaxic coordinates relative to bregma (Franklin and Paxinos, 2007) as shown in Figure 9. Specific targets and naming convention included the hippocampus at HAL (hippocampal anterior left), HAR (hippocampal anterior right), HPL (hippocampal posterior left), HPR (hippocampal posterior right), HVL (hippocampal ventral left), and HVR (hippocampal ventral right) to provide differential measurements of the hippocampal LFPs. Stainless steel screws measuring ECoG referentially are at EFL (EcoG frontal left), EFR (EcoG frontal right), EAL (EcoG anterior left), EAR (EcoG anterior right), EPL (EcoG posterior left), and EPR (EcoG posterior right). Screws at EFL/EFR were used as ground/RE.

The recording system can provide up to 16 channels for data recording. We implant various types and numbers of electrodes based on the specific needs of each experiment; as a result, each animal's implantation may have slight variations. Rats were prepared using the tetanus toxin (TeTX) model, which was first described by Roux and Borrell (Jefferys and Walker, 2006), and has been used to induce seizures mostly in mice and rats (Mellanby et al., 1977). Researchers have worked extensively with TeTX as a model for temporal lobe epilepsy and have characterized both the mechanisms of action of the toxin as well as seizure development and progression (Jefferys et al., 1995; Jefferys and Walker, 2006; Sunderam et al., 2006, 2009; Sedigh-Sarvestani et al., 2014). Usually, after the toxin injection, the animal will start having spontaneous seizures within 10 d. The procedure for TeTX injection and electrode implantation has been previously described (Sedigh-Sarvestani et al., 2014). In short, to induce spontaneous seizures, 10–13 ng of TeTX (Santa Cruz Biotechnology, CAS 676570-37-9) dissolved in 1.3 μl PBS mixed with 2% bovine serum albumin were injected into the rat's left ventral hippocampus (HVL, AP −5.15, ML −5.35, DV −6.1 mm) through a 30 gauge flexible cannula over 15 min, which was then left in place for another 30 min for tissue relaxation. Depth electrodes were implanted in the hippocampus (thalamus or medial septum), while ECoG screw electrodes were placed in the cortex. If oxygen sensing was implemented, hippocampus depth electrodes targeting HAL and HVL would be removed; instead, the WE was implanted to target the hippocampus (HAL), and the custom-made Ag/AgCl RE was placed in the cortex along the same trajectory that was used for toxin injection (HVL), minimizing further damage to the cortex. Depth electrodes, ECoG screw electrodes, WE, and RE were secured in place on the skull and electrically isolated via dental cement. ECG lead electrodes were implanted in the precordium. All leads are connected to the recording system and encapsulated within the 3D-printed head mount. After surgery, rats were returned to individual standard autoclave-ready cages with *ad libitum* access to food and water and maintained at a 12 h light/dark cycle with lights on between 7 A.M. and 7 P.M. We allowed a 7 d postsurgery recovery before initiating recordings. Note that each animal implantation has slight differences based on the experimental purpose, and animal implantation details will be listed along with the circuit diagrams and build information in the data-sharing repository.

### Data acquisition and analysis

After postsurgery recovery, chronic 24/7 continuous data recordings were conducted. During the continuous data recording periods, rats can move freely in the cage. Activities such as adding food, changing water, and cleaning cages were conducted without interrupting the recordings. In Movie 1, we demonstrate a technique for replacing an old cage with a clean one featuring food and fresh water. Briefly, by placing two cages side by side and moving the cage dome to the center, the rat can jump into the new cage on its own while recordings are still ongoing.

**Movie 1.** Cage changing demonstration. A technique to change the old cage with a clean one with food and fresh water. The rat moves from the old cage to the new one, and the recording continues without interruption. [View online]

The data sampling rate was set to 1,000 Hz. Recorded data include LFPs from µRC electrodes targeting the hippocampus, ECoG from screw electrodes over the cortex, ECG recordings, three-axis head acceleration signal, and tissue oxygenation signal using the CPA, and synchronized video. Data are processed offline using custom-written MATLAB (MathWorks) programs for rereferencing, filtering, spectral analysis, and behavior annotation. Unless otherwise indicated, ECoG and LFP recordings are bandpass filtered at 0.5–125 Hz to highlight field potential and seizure dynamics. Average power spectrum density is calculated with a Welch-average 2,048 window after application of the 0.5–125 Hz bandpass filter. Spectrograph and mean square root (MS) power of acceleration are calculated using a 1 s window after 2–125 Hz bandpass filtered. Seizure detection: seizures were detected by a stereotypical increase in spectral power that typically initiates with a sentinel spike followed by a burst of 9–16 Hz hippocampal spikes (Finnerty and Jefferys, 2000), spreads through the cortex, and ends with a sharp decrease in spectral power. For practical purposes, we count only seizure events that are longer than 10 s and spaced apart by at least 10 min. Spreading depolarization detection: SD detections were done from depth electrode measures in the hippocampus referenced to a cortical screw electrode. SD events were detected from signals low-pass filtered with a cutoff at 0.5 Hz, with onset defined by a downward crossing of a 7.5 mV threshold in the signal with respect to the value 3 s before. SD offset was then detected by an upward crossing of a threshold defined as 2 mV above the SD onset potential. Cardiac RR interval measurement: for times when the cardiac signal is free of movement/chest muscle artifact, cardiac ventricular contractions were detected by first bandpass filtering the cardiac signal from 15 to 125 Hz and then locally finding the peak times of the R wave. RR intervals were then computed as the first difference in R peak times. State of vigilance vlassification: SOV was marked as one of the three states—REM state characterized by a spectral peak in the theta (4–7 Hz) frequency band of hippocampal LFP and by an absence of acceleration except during brief muscle twitches; NREM state characterized by maximal power in the delta (0.5–4 Hz) frequency band and by an absence of acceleration; and wake state characterized by the accelerometer activity (Sunderam et al., 2007; Bahari et al., 2021). In vivo CPA response current was low-pass filtered at 1 Hz and then downsampled from 1 kHz to 20 Hz.

## Results

Data were collected from 27 TeTX epileptic rats (21 males and 6 females) and stored on the NAS. We present sample recordings to demonstrate the use of the EMU for synchronized multimodal monitoring tasks capable of studying seizure and SD events.

### Recording example conducted during normal SOV

**Figure 10.**
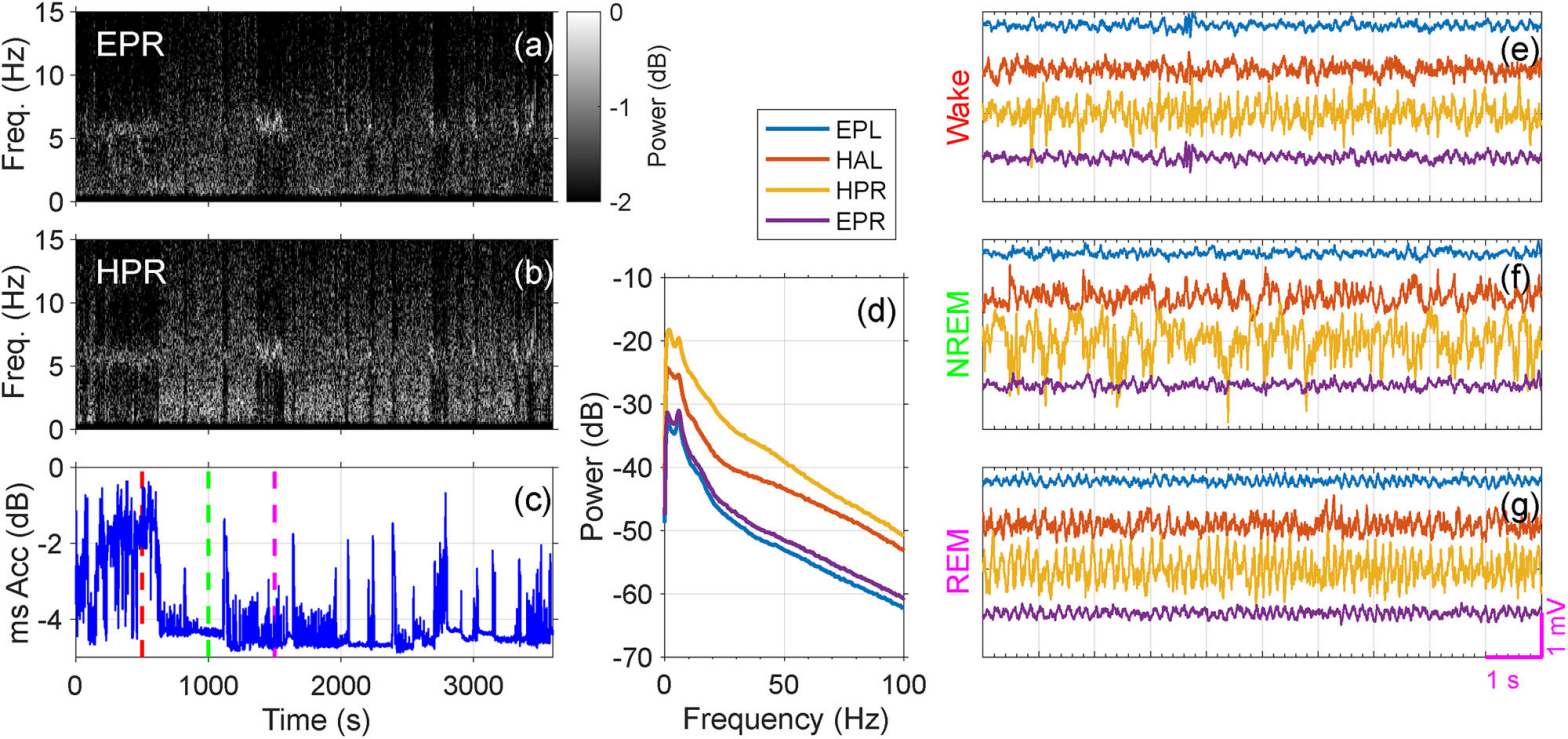
Recording example demonstration conducted during normal SOV. A 1 h recording episode conducted during normal SOV with LFP recordings from the hippocampus depth electrodes, ECoG screws, and head acceleration data. ***a***, The spectrograph for EPR. ***b***, The spectrograph for HPR. ***c***, The mean square root power of head acceleration. ***d***, The power spectrum averaged over 1 h from the EPL, HAL, HPR, and EPR channels, showing that no 60 Hz noise power is detectable (note that no notch filter has been applied). ***e–g***, Example traces of 10 s each of wake, NREM, and REM from the EPL, HAL, HPR, and EPR channels, with the corresponding times marked by color in (***c***).

Neurophysiological studies have demonstrated that sleep–wake states are predominantly characterized by EEG and electromyogram and have been described as discrete states with fast-switching mechanisms (Saper et al., 2005; Datta and Maclean, 2007; Sakai, 2011). Shown in Figure 10 are examples from a 1 h recording episode conducted during a normal SOV with LFP recordings from the hippocampus, ECoG, and head acceleration data. These data were taken ∼14 h before this animal's first seizure event. Spectrograms from ECoG (EPR) and hippocampal LFP (HPR) are presented in (a) and (b), while (c) displays the mean square root power of head acceleration (Acc) for the hour. During the wake state, we observed increased Acc signals consistent with head and body movement, compared with other states (Sunderam et al., 2007). In the NREM state, there was a noticeable increase in delta band power in EPR and HPR, accompanied by a decrease in Acc. During the REM state, we recorded an increased theta band power in EPR and HPR, along with slightly elevated Acc, likely due to brief muscle twitches. The recording system features a high input impedance, paired with the low impedance of the µRC electrode (Shanmugasundaram and Gluckman, 2011), allowing for high-fidelity recordings.

The spectral power averaged over the entire hour for ECoG recordings (EPL, EPR) and hippocampal LFP recordings (HAL, HPR) is shown in (d). Spectra were computed after using a 0.5–125 Hz bandpass filter to accommodate 2 s (2,048 point) windows. Note that no power-line noise pickup is evident in these signals, even though no notch filter was applied.

Example traces illustrating 10 s durations of Wake, NREM, and REM states from EPL, HAL, HPR, and EPR are presented in (e–g). The selected times are marked from the associated colored dotted lines in (c). These signals are consistent with what is expected for these different states. Notice that the REM-related hippocampal theta from this epileptic brain is more angular than from a normal hippocampus, as has been previously observed (Sedigh-Sarvestani et al., 2014).

### Local oxygenation measures using CPA conducted during normal SOV

**Figure 11.**
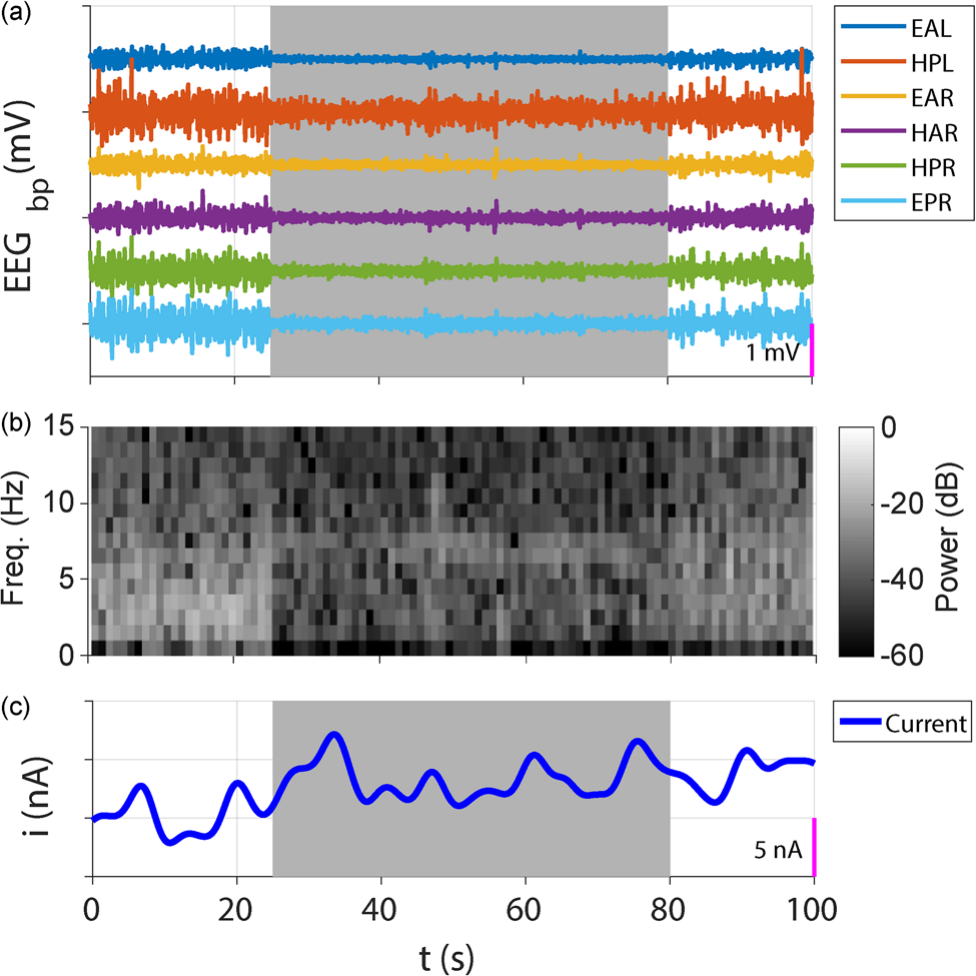
Local oxygenation measures using CPA conducted during normal SOV. A 100 s recording episode conducted during NREM to REM sleep transition from the hippocampus depth electrodes, ECoG screws, and the oxygen-sensing WE. ***a***, Band-filtered ECoG and hippocampal LFP measurements. ***b***, Spectrogram of channel HPR. ***c***, Current response of the CPA from the oxygen-sensing WE. The gray block marks the REM sleep state.

The level of tissue oxygenation provides information related to the balance between oxygen delivery, oxygen utilization, tissue reactivity, and morphology during physiological conditions (Ortiz-Prado et al., 2010). CPA is a well-established method for in vivo oxygen sensing. Shown in Figure 11 is a 100 s multimodal recording during an episode that includes transitions from NREM to REM then back to NREM sleep. Panel (a) shows the bandpass-filtered LFP from the hippocampus (HPL, HAR, and HPR) and ECoG (EAL, EAR, and EPR). The spectrogram from the depth electrode (HPR), highlighting the theta band activity characteristic of the REM sleep state, is shown in Panel (b). The current response of the CPA from the oxygen-sensing WE is shown in Panel (c). The gray blocks in (a) and (c) indicate the periods of REM sleep. The CPA current slightly increased during REM, which should correspond to increased tissue O_2_ during this period.

**Figure 12.**
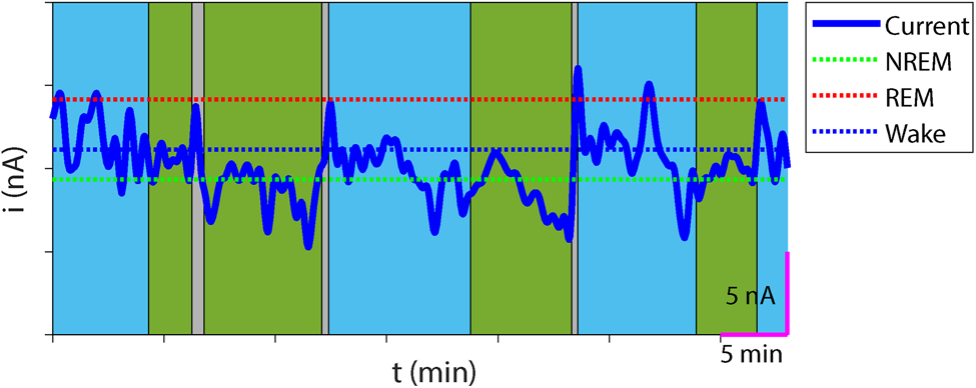
Example of average CPA response current recording conducted during normal SOV. Nearly 1 h (55 min) out of a 2 d CPA current response recording conducted during normal SOV. During this time, the animal experienced multiple episodes of wake (blue block), NREM sleep (green block), and REM sleep (gray block). The red-dotted line represents the mean current value from 73 REM sleep states over 2 d of recordings, the green-dotted line represents the mean current value of 167 NREM sleep states, and the blue-dotted line represents the mean current value of 220 wake states. SOV bouts that started or ended within 1 min of a seizure or SD event were excluded.

Shown in Figure 12 is the average tissue oxygen dynamics measured via CPA over nearly 1 h (55 min) in the same animal. This period was selected due to the absence of seizures and included multiple sleep/wake cycles, which are labeled as REM (gray block), Wake (blue block), and NREM (green block) in the hypnogram. The average current within each state is indicated red for REM (*n* = 73), green for NREM (*n* = 167), and blue for wake (*n* = 220). The averages were taken from 2 full days of measurement and conditionally averaged over SOV bouts that started/ended at least 1 min from any seizure or SD event.

We find a consistently increased CPA measure from NREM to REM sleep and a decreased CPA measure from REM sleep to either NREM sleep or wakefulness. The increased CPA measure during REM sleep indicates a higher oxygen level, likely due to an increased cerebral blood flow since REM sleep is characterized by increased neuronal activity, metabolic demand, and cerebral blood flow, leading to greater oxygen delivery. The decreased CPA measure during NREM sleep suggests a lower oxygen level, as NREM sleep is associated with reduced neuronal activity, metabolic demand, and cerebral blood flow. These results of increased CPA measure during REM sleep and decreased CPA measure during NREM sleep are consistent with earlier studies (McHugh et al., 2011; Dash et al., 2012) that measured brain tissue oxygen levels in rats using CPA.

### Example of abnormal cortical discharges preceding altered cardiac function

**Figure 13.**
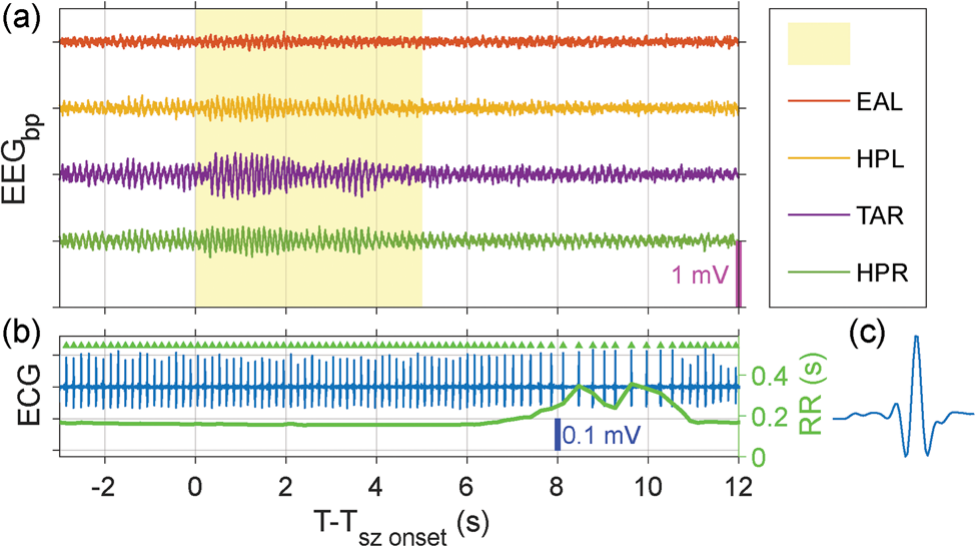
Example of abnormal cortical discharges preceding altered cardiac functions. A recording episode of abnormal fluctuations in brain activity preceding altered cardiac function, as observed from the LFP recordings in the hippocampus (HPL, HPR) and the anterior right thalamus (TAR), as well as from the ECoG (EAL). ***a***, Bandpass-filtered ECoG measure along with hippocampal and thalamus LFPs, highlighting an abnormal 7–8 Hz oscillation with yellow block. ***b***, Low-pass–filtered ECG heart rate measurement, marked in blue, while the point-by-point RR interval (RRI) is marked in green, with *y*-axis on the right. ***c***, Zoom-in view of one heart pulse morphology.

Abnormal fluctuations in brain activity can subtly modify cardiac function long before microscale instabilities propagate into a significant seizure (Bragin et al., 2000; Schevon et al., 2008; Stead et al., 2010). The cortically induced disturbances of cardiac rhythm maybe responsible for making the network more susceptible to more seizures and vulnerable to their effects (Lathers et al., 1987; Schuele et al., 2007). In the study (Bahari et al., 2018), the authors identified a brain–heart biomarker for epileptogenesis in a murine model. This biomarker is characterized by occasional long cardiac RR intervals that precede fluctuations in cortical activity, detectable weeks to months before the animals experience their first seizures. In our TeTX model, we observed similar patterns in three animals that had implanted pericardial electrodes. An example of abnormal fluctuations in brain activity affecting cardiac function is shown in Figure 13, as observed from the LFP recordings in the hippocampus (HPL, HPR) and the anterior right thalamus (TAR), as well as from the ECoG (EAL). Panel (a) shows bandpass-filtered ECoG measurement alongside hippocampal and thalamic LFPs (EEG_bp_), highlighting an abnormal oscillation at 7–8 Hz that lasts ∼5 s with a yellow block. Approximately 5 s later, the low-pass–filtered ECG heart rate measurement shows a cardiac arrhythmia event with substantially long RR intervals, as shown in Panel (b), marked in green, with the *y*-axis on the right side. The abnormal oscillations observed in this study were more sustained than the short (<2 s) higher-frequency components reported by Bahari et al. (2018). Generally, the cardiac measures were significantly affected by muscle and movement artifacts, especially during seizures; therefore, observations of cardiac arrhythmia were limited to periods when the animal was relatively still, such as during sleep. A complete analysis of these dynamics will require future studies that utilize improved cardiac electrode design and placement techniques.

### Recording example of a spontaneous seizure and seizure-associated spreading depolarization

Spreading depolarization/depression (SD) found and named by Leao in 1944 in the cortex of epileptic rabbits (Leao, 1944) is characterized by a large negative DC shift in tissue potential amplitude of 35 mV or more and duration lasting for ∼30–60 s (Grafstein, 1956; Kraig and Nicholson, 1978; Somjen et al., 1992). SD is reported to be closely associated with epilepsy and epileptogenesis (Dreier et al., 2012; Eickhoff et al., 2014). DC sensitivity and large dynamic range and bit depth are critical for being able to span the dynamics of normal field potentials, seizures, and spreading depolarization in a single recording.

**Figure 14.**
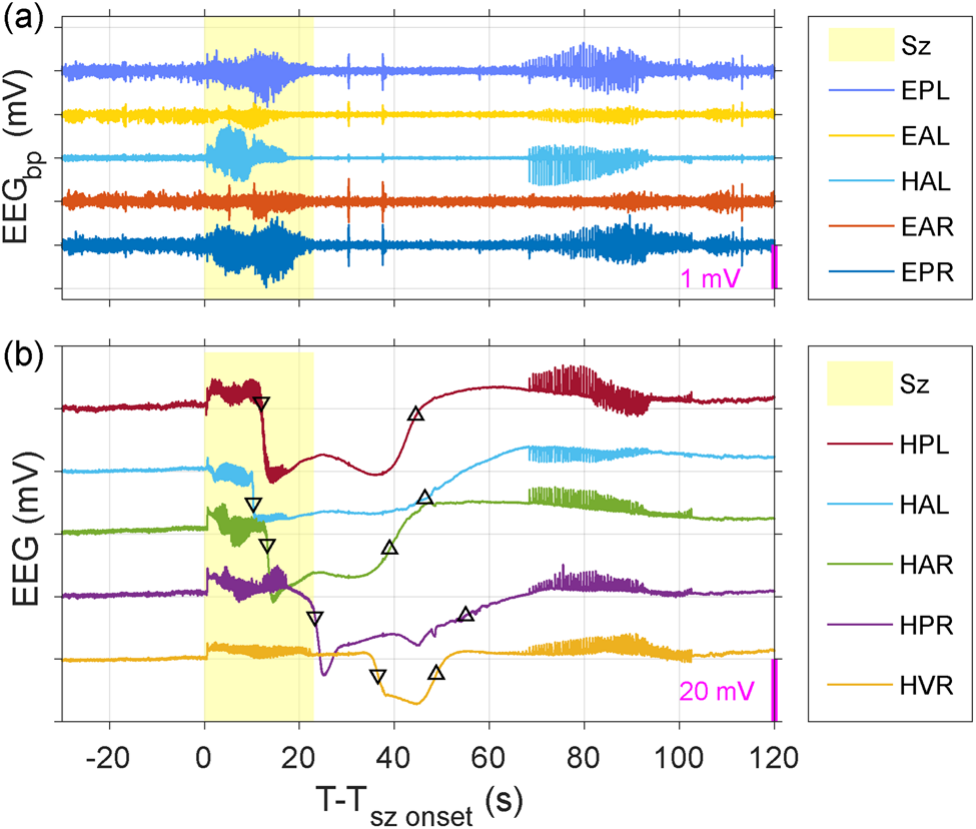
Example of seizure and seizure-associated spreading depolarization event. A recording episode showing a transition from normal SOV to seizure followed by seizure-associated spreading depolarization (SD) event. ***a***, Bandpass-filtered ECoG (EPL, EAL, EAR, and HAL) along with hippocampal LFP measurements (HAL). ***b***, Low-pass–filtered hippocampal LFP measurements (HPL, HAL, HAR, HPR, and HVR), along with interaction with seizure-associated SD. See also Extended Data Figure 14-1 for an example of seizure and seizure-associated spreading depolarization clusters and Extended Data Figure 14-2 for an example of seizure-related DC shift. The ictal period is marked in yellow, and triangles indicate the SD propagation downward and upward dynamics.

In our TeTX model, we found frequent instances of spontaneous seizures and seizure-associated SD events. One example of a seizure and seizure-associated SD event is shown in Figure 14. Panel (a) shows bandpass-filtered ECoG (EPL, EAL, EAR, and HAL) along with hippocampal LFP measurements (HAL; EEG_bp_), with the ictal period marked in yellow. Panel (b) shows low-pass–filtered hippocampal LFP measurements (HPL, HAL, HAR, HPR, and HVR; EEG) with the seizure marked in yellow, along with interaction with seizure-associated SD, with triangles indicating the SD propagation downward and upward dynamics. We found that following ictal onset, seizures that evolve into SD undergo a silent period, followed by recovery after-discharges, i.e., spreading convulsions. The DC measurements clarify that the depressed EEG activity and the later after-discharges are seizure-associated SD events, as well as recovery from them. The negative DC shift of SD can be of order 35 mV or more, and there is a propagation pattern from HPL to HAL on the left hippocampus and from HAR to HPR and to HVR on the right hippocampus. This phenomenon would not be observable without DC-sensitive multiple-channel recordings.

10.1523/ENEURO.0057-25.2025.f14-1 Figure 14-1 **Example of seizure and seizure-associated spreading depolarization clusters.** A recording episode showing three pairs of seizures and seizure-associated SD events clustered together over a duration of 1200 seconds. **(a)** Band pass filtered ECoG along with LFP measurements. **(b)** Low pass filtered hippocampal LFP measurements. Download Figure 14-1, TIF file.

10.1523/ENEURO.0057-25.2025.f14-2 Figure 14-2 **Example of seizure related DC-shift.** A recording episode showing a transition from normal SOV to seizure with a DC-shift. Low pass filtered hippocampal LFP measurements. Download Figure 14-2, TIF file.

From the animals recorded with this EMU, we counted 3,368 seizures from 16 animals, of which 11 animals had 773 SD events. Animals with too many seizure-SD clusters (e.g., loki011, 014) were excluded based on the seizure counting criteria described in Materials and Methods. One example of seizure and seizure-associated SD clusters is shown in Extended Data Figure 14-1. Panel (a) shows bandpass-filtered ECoG (EAL) along with hippocampal (HPL, HAR) and anterior right thalamus (TAR) LFP measurements (EEG_bp_), with the ictal period marked in yellow. Panel (b) shows low-pass–filtered hippocampal (HPL, HAR, HPR, and HVR) and TAR LFP measurements (EEG) with the ictal period marked in yellow, showing three pairs of seizures and seizure-associated SD events clustered together over a duration of 1,200 s, with up and down triangles indicating the dynamics of SD propagation.

One notable point to consider when studying seizure-associated SD is that both seizures and SD events result in slow potential (DC) shifts in LFP recordings. However, their mechanisms and characteristics are distinct. Compared with SD, seizures produce a smaller DC shift, typically <5 mV, which coincides with ongoing hypersynchronous spiking activity (Caspers et al., 1987; Ikeda et al., 1999; Herreras, 2016). Instead of a breakdown of ion homeostasis, the DC shift associated with seizures arises from sustained synaptic and glial currents, extracellular potassium (K^+^) accumulation, and changes extracellular space volume. In our TeTX model, seizure-generated DC shifts are also observed, as illustrated in Extended Data Figure 14-2 but with much smaller amplitudes and no spatial propagation. In low-pass–filtered recordings of hippocampal LFPs (HPL, HPR, and HAR; EEG), a seizure-generated DC shift shows an amplitude of ∼5 mV, which is significantly smaller compared with the SD-related DC shift, and no propagation pattern is observed.

### Semiology demo with synchronized EEG, ECG, and video recordings

Synchronized multimodal recordings, particularly EEG video combined with ECG, are essential for comprehensive seizure investigation in both clinical and preclinical settings. In humans, video EEG remains the gold standard for diagnosing epilepsy, enabling precise correlation between electrographic events and behavioral manifestations (Fisher et al., 2014), while synchronized ECG recording captures seizure-related autonomic alterations, such as heart rate variability, arrhythmias, and peri-ictal tachycardia or bradycardia, which are critical for understanding mechanisms underlying SUDEP (Surges et al., 2009; Ryvlin et al., 2013; Myers et al., 2018). In animal models, synchronized EEG video and ECG similarly allow simultaneous assessment of neural activity, behavior, and autonomic physiology, enabling precise characterization of cortical and subcortical seizure dynamics alongside ictal cardiorespiratory changes (Kandratavicius et al., 2014; Aiba and Noebels, 2015; Levesque et al., 2018).

In the EMU, we generate two different file streams (i.e., electrophysiological/electrochemical recordings and video recordings) from individual single-board computers. The starting time of the data in each file is reflected in the file names. Actual synchronization then relies on the computer clocks, which are synchronized to a network-attached time server. To separately double-check that the two data streams are synchronized, we compute the cross-correlation between a video-based actigraphy measurement, calculated from the average of the absolute value of the first-time derivative of pixel intensities, to an actigraphy from the head-mounted accelerometers, both measured on 1 s windows and high-pass filtered at 10 mHz. For data measured after implementation of the time server, these correlation functions consistently peak at 0 ± 1 s, confirming synchronized electrophysiological/electrochemical recordings and video recording.

Movie 2 presents a 300 s semiology demo with ECoG, ECG, hippocampus LFP measurements, and synchronized video recording, showing progression from normal SOV to seizure and then to seizure-associated SD event. On the left, the recorded freely moving rat in its cage is shown in Panel (a), while ECoG, ECG recordings, and hippocampus LFP measurements appear on the right. During the seizure, the bandpass-filtered ECoG and hippocampus LFP (EEG_bp_) in Panel (b) reveals high-amplitude poly-spike bursts across multiple electrodes [EAL, EAR, HPL, HAL, VL-TL (ventrolateral thalamus), HAR, and HVR]. Simultaneously, the video captures distinct behavioral signs: the rat suddenly freezes, rears, exhibits forelimb clones, loses balance, and falls. This is followed by a period of postictal immobility. Even though the ECG recording (c) was affected by the rat's seizure-induced movements, it still demonstrated detectable cardiac spikes. Notably, in low-pass–filtered hippocampus LFP recordings (EEG) shown in Panel (d), a seizure-associated SD event is clearly observed, suggesting it follows or interacts with the seizure activity. Note that in these recordings, the hippocampal measurements are referenced to a common cortical screw electrode. While the SD event is localized to the hippocampus, the potential measurements mix between the cortical and hippocampal fields.

**Movie 2.** Seizure to spreading depolarization semiology demo. A 300 s semiology demo with ECoG, hippocampus LFP measurements, ECG, and synchronized video recordings, demonstrating a normal SOV to seizure, and to seizure-associated SD transition. The video (***a***) on the left shows a freely moving rat under recording. On the right, (***b***) bandpass-filtered ECoG (EAL and EAR), along with LFP measurements (HPL, HAL, VL-TL, HAR, and HVR). ***c***, Bandpass-filtered ECG recordings. ***d***, Low-pass–filtered hippocampal LFP measurements (HAL, HAR, and HVR). The demo starts from 60 s before seizure onset (*t* = 0), signals propagate with real-time updates displayed in ***b***, ***c***, and ***d***, with synchronized video (***a***) showing the rat's behavior. Scale bar between grid lines (10 mV/1 s) indicates both the signal amplitude and the time alignment between the signal traces and the video. [View online]

## Discussion

We present a new EMU that enables chronic, 24/7, high-fidelity, synchronized multimodal recordings from multiple animals at low cost (<$1,000 per rig). Each rig is compatible with standard racks, uses autoclave-ready cages without modification, and can be easily scaled. The demonstrations confirm that the EMU meets the design criteria outlined in the Introduction section, integrating high-resolution electrophysiology, synchronized behavior, and electrochemical sensing to provide a robust platform for continuous home-cage monitoring in epilepsy studies of freely moving animals.

The hippocampal (HC) LFP recordings (Fig. 10) validate the system's ability to capture both the high-frequency components of seizures and the large, slow DC shifts associated with SD. The transition from AC-coupled seizure activity to DC-sensitive depolarization highlights the importance of wide bandwidth, high input impedance, and high-resolution analog-to-digital conversion. The large dynamic range ensured that microvolt-level oscillations and millivolt-scale DC shifts were faithfully recorded without saturation, confirming the system's ability to capture both seizures and SDs. Multichannel recordings further demonstrated the capacity to track the spatiotemporal propagation of SDs within the HC, from local foci on the left HC to distant regions on the right HC. Synchronized video provided precise behavioral correlates of electrographic activity, with alignment to LFP/ECoG enabling reliable identification of seizure semiology and behavioral state transitions (Movie 2).

Compared with commercially available systems, which often have limitations in channel count, sampling rate, and recording duration, while also being prohibitively expensive, our EMU offers a more powerful and cost-effective solution. Each rig supports up to 16 channels, enabling synchronized recordings that include LFP, ECoG, ECG, head acceleration, and time-synchronized video. A built-in EI allows monitoring of local tissue chemistry. In addition, the EMU offers DC sensitivity, sampling rates up to 16 kHz, broad bandwidth, and low baseline noise, preserving high-fidelity signals across diverse applications. These capabilities are delivered at a fraction of the cost per channel compared with commercial systems, making the EMU a practical and scalable option for high-channel, continuous, multianimal studies.

The EMU subsystems can be used individually or in combination, depending on application needs. For example, acceleration parameters have long been employed to study TBI pathology, guide safety equipment design, and establish injury thresholds (Mayer et al., 2021). Acceleration measures have also been used to identify behavioral modes (Chen et al., 2019; McNamara et al., 2020; Mayer et al., 2021; Maekawa et al., 2022). In our EMU, three-axis head acceleration recording allowed monitoring of head-bobbing, freezing, and full-body convulsions during seizures and postictal periods. The commutator and isolation system in our EMU enables recordings in freely moving animals. Many rodent epilepsy studies rely on anesthesia, which can alter brain function. Recording neural activity in freely moving mice is a powerful and flexible technique for dissecting the neural circuit mechanisms underlying pathological behavior (Harris et al., 2017). However, cable connections for data transmission restrict the animal's behavior. Wireless recording systems have been applied in freely moving animal studies (Kramer and Kinter, 2003; Kadam et al., 2010; Zayachkivsky et al., 2015; Lundt et al., 2016). However, these systems face major challenges, including short battery life for long-term continuous monitoring, low and unstable data rates with limited channel counts, and very high costs. Our system overcomes these issues with a commutator that operates standalone without counterbalanced bars or bearings and provides direct USB connectivity with high and stable data rates with isolation features for human compatibility, making it suitable for brain–computer interface applications.

Electrochemical methods are powerful tools in neuroscience (Borland and Michael, 2007), providing complementary signals such as tissue oxygenation alongside behavioral, electrophysiological, and optical measurements. Electrochemical oxygen monitoring remains the “gold standard” (Stone et al., 1993; Vaupel et al., 2007). Adams’ early slow-scan voltammetry experiments have given way to high temporal resolution voltammetry measurements at micron- and submicron-scale electrodes (Johnson, 2013). Different voltametric and amperometric techniques have been combined with electrophysiology and enzyme-mediated biosensors (Wilson and Johnson, 2008), expanding the tools available to investigators (Johnson, 2013). In vivo electrochemistry has grown steadily and has been applied for neurotransmitter sensing. Using FSCV, norepinephrine and dopamine sensing (Park et al., 2011) has been done in rats, and norepinephrine sensing has been reviewed in detail by Park et al. (2018). Long-term in vivo electrochemistry enables real-time monitoring and measurement of brain metabolites (Lowry et al., 2010).

Using the built-in EI in the EMU, we demonstrated in vivo oxygen sensing using CPA on freely moving animals, coupled with electrophysiology recordings (Figs. 11, 12). While CPA is widely used to measure local tissue oxygenation, it assumes a constant oxygen diffusion coefficient and cannot decouple the effects of local oxygen concentration and the oxygen diffusion coefficient in the CPA measure (Subczynski and Swartz, 2005). This limitation becomes critical during events such as SD, when diffusion properties change. With the integrated function generator, our EMU enables the development of innovative oxygenation sensing methods suitable for periseizure and peri-SD states. Moreover, our EMU shows potential for studying neurovascular coupling and metabolic dynamics in freely moving animals by combining in vivo *PO*_2_ measurements with electrophysiology and neural imaging.

## Synthesis

Reviewing Editor: Catherine Schevon, Columbia University Decisions are customarily a result of the Reviewing Editor and the peer reviewers coming together and discussing their recommendations until a consensus is reached. When revisions are invited, a fact-based synthesis statement explaining their decision and outlining what is needed to prepare a revision will be listed below. The following reviewer(s) agreed to reveal their identity: Elliot Smith, Hongkun Zhu.

The manuscript provides a technical solution for recording multimodal neurophysiology and behavior in a freely moving rodent model of epilepsy (tetrodotoxin). The description of the device is comprehensive and thorough. The authors recap results from two studies using the system reported in the manuscript. Unfortunately, the authors don't reference those studies in the appropriate place, which makes the results presentation seem unnecessarily cursory. Both reviewers agreed that this manuscript would be an important addition to the literature after minor revision.

Major critiques:

1. The manuscript states that SDs were either referenced to a bipolar pair or to an ECoG electrode. How did the different referencing schemes affect the detection or properties of SDs?

2. Figure 10 is really the main data figure, so should really be presented clearly. The lines in Figure 10 are too thick, so that the seizure dynamics are obscured and only the signal density can be seen. Can those lines be made thinner so that the finer scale seizure dynamics are visible? There is no x-axis unit label in Figure 10. Instead of plotting the traces at different voltages, as the figure currently does, It might be more accurate to replace the y-axis with a scale bar.

3. Are citations to the other two studies removed for anonymity? If not, they should be added when they are referred to at the beginning of the results. I assume that more detailed results are in the other papers, but was unable to assess that directly, so the results presented here seem superficial, which makes all of the claims in the results seem overstated. The claim on line 291 is clearly overstated and could be cherry-picked without included quantification across seizures. The cardiac and oxygenation results comes across similarly. If the authors are just including these examples to show the functionality of the system, that's fine, but they shouldn't overstate physiological claims in this work. If these claims are based on rigorous quantification from other studies, they could be included with adequate citation. What would actually really support the claims in the paper would be providing summary statistics and quantification of the effects the authors make claims about across all observations that their cool technology provides.

4. More information about the fidelity of the video and behavioral monitoring would strengthen the manuscript, since semiology is one of the most important aspects of seizure monitoring in the clinic.

5. One key advantage of this long-term monitoring system is its ability to support multi-modal data recording simultaneously. A more illustrative figure showing all reported modalities aligned together is favored-such as screenshots of the rat's behavior, EEG or spectrogram from sample channels, head acceleration, and oxygen dynamics. While Figure 9 presents simultaneous spectrogram and head movements, it could be more informative by incorporating oxygen dynamics and example screenshots of different states (e.g., sleep/awake), if these data are available.

6. In line 295, the authors claim to have observed long cardiac RR intervals similar to those reported in the cited paper, "A Brain-Heart Biomarker for Epileptogenesis" (Bahari, Ssentongo et al., 2018). However, the example in Figure 11 shows abnormal 7-8 Hz oscillations across subcortical regions, whereas the cited paper describes high-frequency activity in the cortical and hippocampal areas. Could you clarify the specific similarities between the findings reported here and those in the cited paper?

Minor critiques:

- Line 39: There appears to be a possessive missing in this clause. The containing sentence is more of a list and could be revised for clarity.

- Line 87: Some commas are missing in this sentence.

- Line 134: More information about the file format could be provided.

- Line 149: The object of 'these' is unspecified.

- Line 197: The Jeffreys citation is incorrectly capitalized.

- Line 275: it seems like there is a word missing in this sentence between 'power' and 'four.'

- In line 250-251, Please specify if an overlap or non-overlapped window is used to calculate PSD and spectrogram.

- Line 229 Fix the typo "100µm" instead of "100um"

- Line 355 Fix the text font and size

- Line 376-377 Fix the text font and size

- Line 414-416 Fix the text font and size

- Figure 9c is missing colormaps for the spectrograms (subplot c). In addition, subplot labeling in the caption is in bold and uppercase, while in the figure, it is in lowercase. Please ensure consistency between the caption and figure.

In addition, the reviewers have requested that the authors make their circuit diagrams and code open source, to permit other groups to make use of this technology.
