# Supplementary figures and images for: A DC-Sensitive Video/Electrophysiology Monitoring Unit for Long-Term Continuous Study of Seizures and Seizure-Associated Spreading Depolarization in a Rat Model
Source: eNeuro. 2026 Jan 14;13(1):ENEURO.0057-25.2025. doi: 10.1523/ENEURO.0057-25.2025 (PMC12834326; doi:10.1523/ENEURO.0057-25.2025)

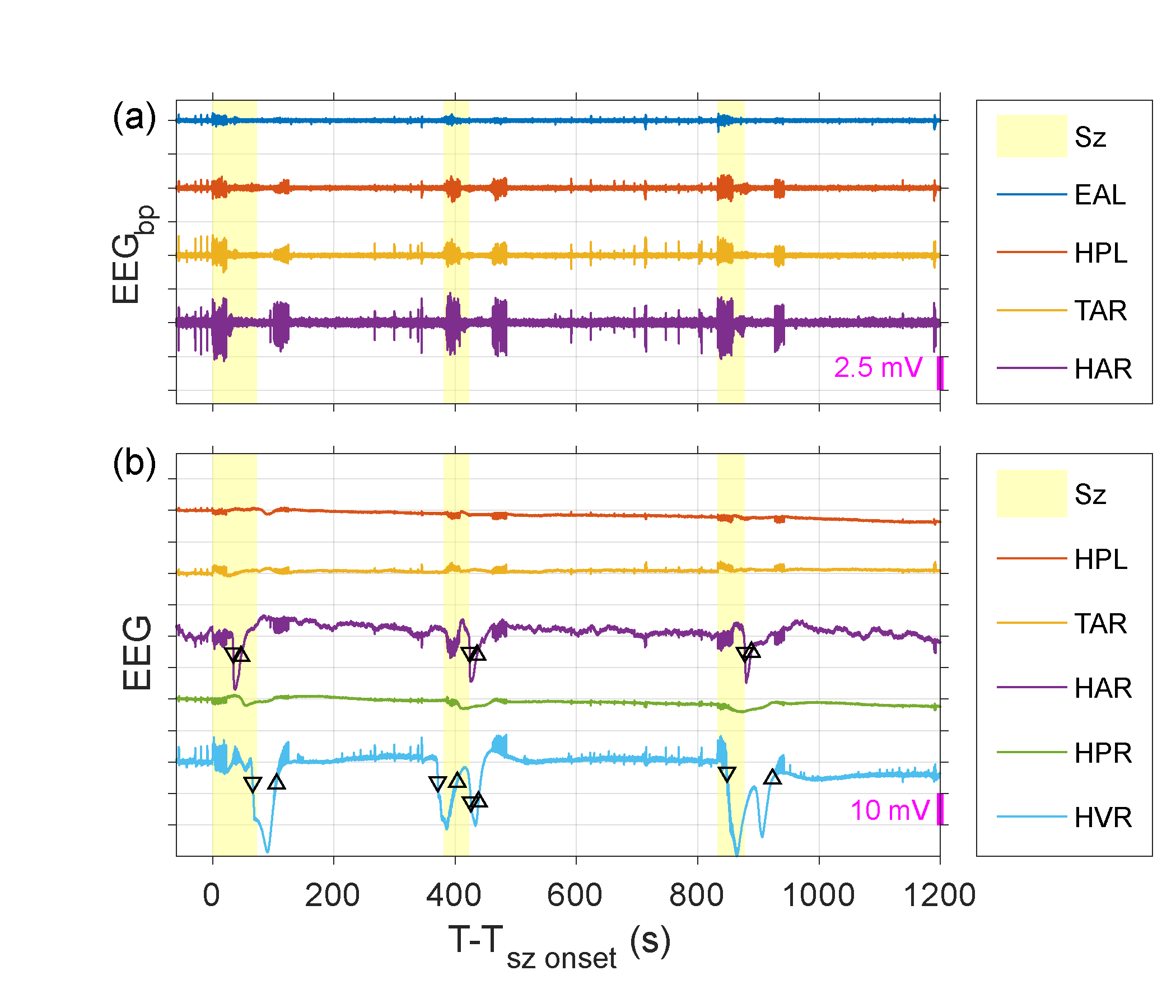

Supplement: Figure 14-1 — Example of seizure and seizure-associated spreading depolarization clusters. A recording episode showing three pairs of seizures and seizure-associated SD events clustered together over a duration of 1200 seconds. (a) Band pass filtered ECoG along with LFP measurements. (b) Low pass filtered hippocampal LFP measurements. Download Figure 14-1, TIF file. [file eneuro-13-ENEURO.0057-25.2025-s001.tif]

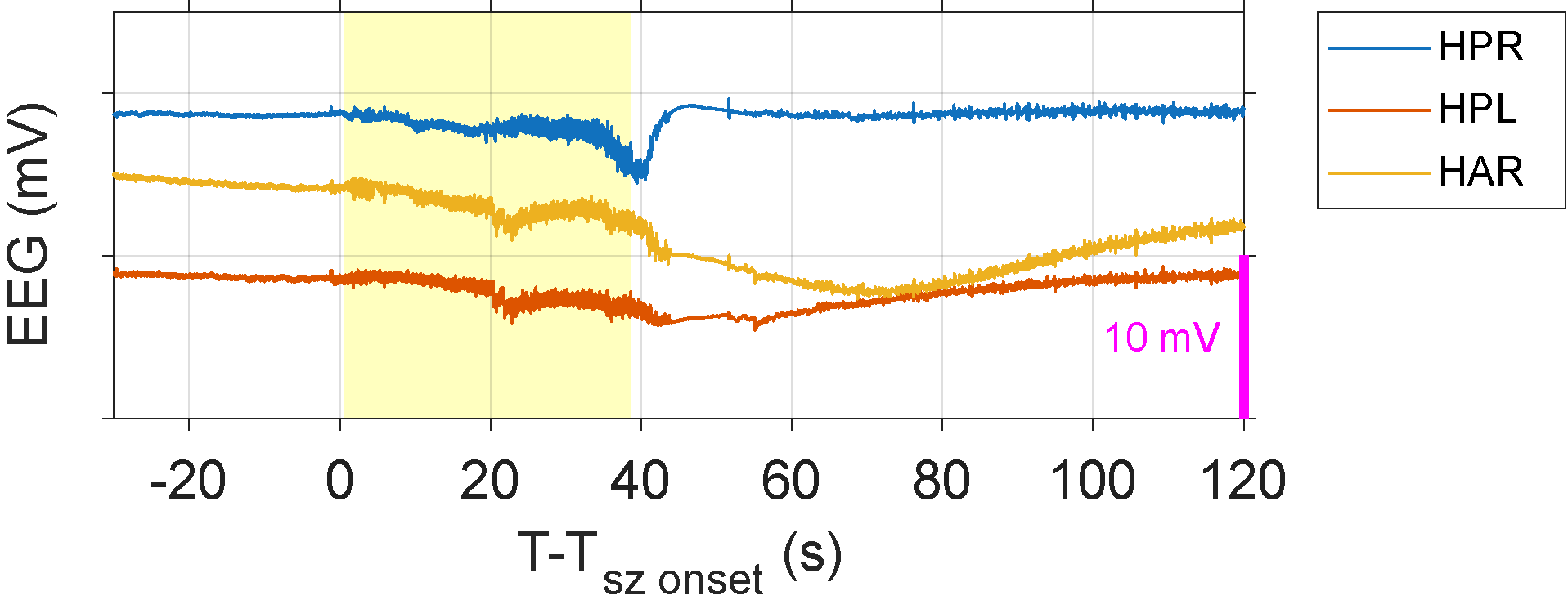

Supplement: Figure 14-2 — Example of seizure related DC-shift. A recording episode showing a transition from normal SOV to seizure with a DC-shift. Low pass filtered hippocampal LFP measurements. Download Figure 14-2, TIF file. [file eneuro-13-ENEURO.0057-25.2025-s002.tif]
